# Qifu Decoction Alleviates Lipopolysaccharide-Induced Myocardial Dysfunction by Inhibiting TLR4/NF-κB/NLRP3 Inflammatory Pathway and Activating PPARα/CPT Pathway

**DOI:** 10.3390/ph18081109

**Published:** 2025-07-25

**Authors:** Lingxin Zhuo, Mingxuan Ma, Jiayi Zhang, Jiayu Zhou, Yuqi Zheng, Aiyin Liang, Qingqing Sun, Jia Liu, Wenting Liao

**Affiliations:** 1 Department of Pharmaceutical Analysis, China Pharmaceutical University, Nanjing 210009, China; lingxinzz@163.com (L.Z.); mmxuan0910@163.com (M.M.); 19850859906@163.com (J.Z.); 15857004688@163.com (J.Z.); yqzheng002@163.com (Y.Z.); 13657746366@163.com (A.L.); sunqingqing123025@163.com (Q.S.); 2 Key Laboratory of Drug Quality Control and Pharmacovigilance, Ministry of Education, China Pharmaceutical University, Nanjing 210009, China; 3 Pharmic Laboratory Animal Center, China Pharmaceutical University, Nanjing 210009, China; liujia944@163.com

**Keywords:** Qifu decoction, sepsis-induced cardiomyopathy, TLR4/NF-κB/NLRP3 inflammatory pathway, PPARα/CPT pathway, fatty acid oxidation

## Abstract

**Background/Objectives**: Sepsis-induced cardiomyopathy (SIC) is a serious clinical disorder with a high death rate. Qifu decoction (QFD) is a renowned traditional Chinese medicine with documented pharmacological actions, such as anti-inflammatory, anti-oxidant and anti-apoptosis activities, and it has good therapeutic effects on cardiovascular diseases. This study aimed to reveal the cardioprotective effects and underlying mechanisms of QFD against SIC. **Methods**: Electrocardiography, histopathological examination, and biochemical indicator determination were carried out to investigate the cardioprotective effects of QFD in the treatment of LPS-induced SIC mice. Metabolomics and network pharmacology strategies were employed to preliminarily analyze and predict the mechanisms of QFD against SIC. Molecular docking and Western blot were further applied to validate the core targets and potential pathways for the treatment of SIC in in vitro and in vivo models. **Results**: It was found that QFD considerably enhanced cardiac function; attenuated myocardial injury; and reduced the serum levels of LDH, CK-MB, IL-1β, and TNF-α by 28.7%, 32.3%, 38.6%, and 36.7%, respectively. Metabolomic analysis showed that QFD could regulate seven metabolic pathways, namely, glutathione metabolism; alanine, aspartate, and glutamate metabolism; arachidonic acid metabolism; glycerophospholipid metabolism; purine metabolism; sphingolipid metabolism; and fatty acid metabolism. Network pharmacology suggested that the anti-SIC effect of QFD may be mediated through the TNF, toll-like receptor, NOD-like receptor, NF-κB, and PPAR signaling pathways. Additionally, 26 core targets were obtained. Molecular docking revealed that active ingredients such as formononetin, kaempferol, quercetin, and (R)-norcoclaurine in QFD had a high affinity for binding to PPARα and TLR4. Further Western blot validation indicated that QFD could regulate the protein levels of NLRP3, TLR4, NF-κB, IL-6, TNF-α, COX2, sPLA2, PPARα, CPT1B, and CPT2. **Conclusions**: This study demonstrates that QFD can alleviate SIC by suppressing the TLR4/NF-κB/NLRP3 inflammatory pathway and modulating impaired FAO through the activation of the PPARα/CPT pathway, highlighting QFD as a promising candidate drug for SIC treatment.

## 1. Introduction

Sepsis is defined as life-threatening organ dysfunction caused by a dysregulated host response to infection, which is the primary cause of death in intensive care units [1]. A significant proportion of patients with sepsis may develop sepsis-induced cardiomyopathy (SIC), which represents the most severe and lethal complication of sepsis and may have a mortality rate of up to 70–90% [2]. SIC is an acute cardiac injury, characterized by impaired left ventricular systolic and diastolic function [3].

Accumulating evidence demonstrates that the pathogenesis of SIC is very complex, involving dysregulated inflammatory mediators, mitochondrial dysfunction, oxidative stress, dysregulated calcium, and endothelial dysfunction [3]. Both the dysregulation of inflammatory mediators and the dysfunction of mitochondria are fundamental pathologies of SIC [3]. Cardiomyocytes are densely packed with mitochondria to fulfil their energy demand. During sepsis, the dramatic inflammatory response can lead to myocardial mitochondrial dysfunction, which may induce serious metabolic disorders, oxidative stress, and, subsequently, myocardial injury and apoptosis [4,5]. Cellular damage further aggravates the effects of inflammation on tissue damage [6]. Thus, inhibiting inflammation and maintaining myocardial mitochondrial homeostasis may be effective therapeutic strategies for SIC. Currently, β-blockers, inotropes, and anti-inflammatory drugs are commonly used clinically to treat SIC [7], but specific therapeutic agents are still lacking. In view of the increasing incidence of SIC, it is urgent to explore new therapeutic drugs for SIC. In recent years, greater progress has been made in the treatment of SIC with traditional Chinese medicine (TCM). For example, Xu et al. found that QX1 attenuated SIC by inhibiting cardiomyocyte apoptosis [8]. Wang et al. reported that xiaochaihu decoction ameliorated LPS-induced myocardial injury, downregulated genes associated with PANoptosis, and could be used to treat SIC [9]. TCM offers new ideas for the treatment of SIC.

Qifu decoction (QFD) originated in *Wei Shi Jia Cang Fang* (Wei’s Family Prescription). It is an ancient prescription comprising Huangqi (Astragali Radix) and Fuzi (Aconiti Lateralis Radix Praeparaia) in a mass ratio of 2:1. The plant names were checked with MPNS (http://mpns.kew.org). In ancient texts, QFD is mainly used to treat qi deficiency, yang deficiency, limb fatigue, continuous sweating, and weak pulse, which is the theoretical basis of traditional Chinese and Western medicine for the treatment of cardiovascular diseases [10]. Modern pharmacological research has found that QFD has anti-inflammatory, anti-oxidant stress, and therapeutic effects on major myocarditis [11,12], premenstrual tension syndrome, and oral ulcers [13]. Moreover, the component herbs of QFD have shown excellent protective effects against heart failure in the laboratory and clinical practice [14,15,16]. At present, there are no relevant research reports on the therapeutic effect of QFD on SIC. As QFD has cardioprotective potential, we hypothesized that QFD could be an efficacious therapeutic strategy for alleviating SIC.

In this paper, we firstly investigated the cardioprotective effects of QFD in the treatment of SIC using an LPS-induced SIC mouse model, and then we preliminarily analyzed and predicted the mechanisms of QFD against SIC by combining metabolomics with a network pharmacology strategy. Based on these results, we further validated the potential pathways and core targets identified for the treatment of SIC in in vitro and in vivo models. It was finally revealed that QFD might alleviate SIC by suppressing the TLR4/NF-κB/NLRP3 inflammatory pathway and modulating impaired FAO through activation of the PPARα/CPT pathway.

## 2. Results

### 2.1. Ingredient Identification of QFD by UPLC-QTOF-MS

A base peak chromatogram of QFD and extracted ion chromatograms of songorine and astragaloside IV in standard solution and QFD in positive ion mode are shown in Figure S1A–C. QFD was standardized to contain 31.2 μg/g songorine and 48.1 μg/g astragaloside IV. Additionally, 50 (in vitro) and 30 (in vivo) compounds were detected and identified in QFD, and specific information on the retention time (t_R_), molecular formula, and mass-to-charge ratio (*m*/*z*) of each compound is summarized in Table 1 and Table S1, respectively.

### 2.2. QFD Alleviated Symptoms of SIC in Mice

**Figure 1:**
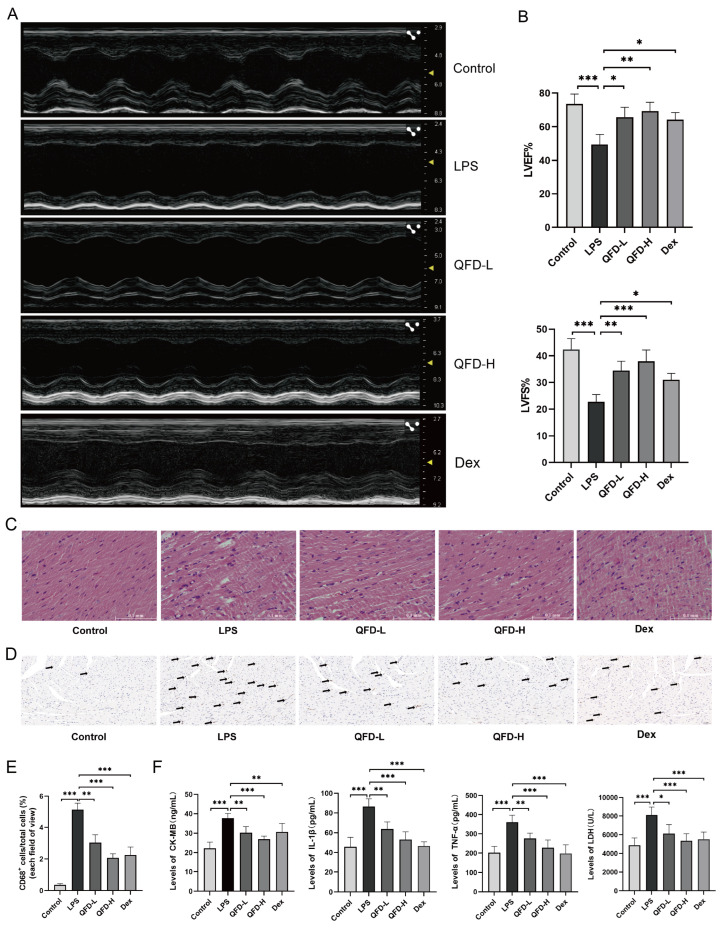
QFD alleviated symptoms of SIC in mice. (**A**) Representative two-dimensional images of echocardiography. (**B**) Left ventricular ejection fraction (LVEF) and left ventricular fraction shortening (LVFS) (n = 5). Mice in QFD-L and QFD-H groups were administered intragastrically with QFD at 3 and 6 g/kg, respectively, for 7 d. Dex group mice were injected intraperitoneally at a dose of 10 mg/kg for 7 d. Other groups were treated with the same volume of physiological saline. Then, 1 h after the last administration, mice in LPS, QFD, and Dex groups were intraperitoneally injected with LPS (10 mg/kg); 10 h later, cardiac function was measured, and blood samples and heart tissues of mice were collected for further analysis. (**C**) HE staining images of myocardium (scale bar = 0.1 mm, original magnification 200×). (**D**) Immunohistochemical images of CD68-positive cells in myocardium (scale bar = 50 μm, original magnification 40×). The arrows point to CD68-positive cells. (**E**) Counts of CD68-positive cells in each field of view (n = 3). (**F**) Levels of IL-1β, TNF-α, CK-MB, and LDH in serum of SIC mice (n= 10–12). Data are expressed as mean ± SD. * *p* < 0.05, ** *p* < 0.01, *** *p* < 0.001.

The echocardiography results indicated that, compared to the control group, left ventricular systolic function (Figure 1A) and LVEF and LVFS (Figure 1B) were obviously impaired in the LPS group mice, indicating that LPS could induce cardiac dysfunction in mice. QFD (3 and 6 g/kg) treatment reversed the LPS-induced decrease in LVEF and LVFS. Histopathological analysis revealed that the myocardial tissue of the LPS-induced mice showed obvious cytoplasmic vacuolation and loose deformation of the cardiac muscle fiber structure. QFD treatment effectively moderated the above morphological changes in the SIC mice (Figure 1C). CD68 is highly expressed in macrophages and is a signal of inflammation in vivo. During sepsis, CD68-positive cells accumulate in cardiac tissues [17]. As shown in Figure 1D,E, there was an evident change in the proportion of CD68-positive cells in the LPS group, which was effectively mitigated by QFD and Dex. Additionally, the serum levels of proinflammatory factors (IL-1β and TNF-α) and markers of myocardial damage (CK-MB and LDH) in the SIC mice significantly increased, indicating that LPS caused serious inflammation and myocardial injury. QFD treatment reduced the serum levels of LDH, CK-MB, IL-1β, and TNF-α by 28.7%, 32.3%, 38.6%, and 36.7%, respectively (Figure 1F).

### 2.3. Metabolomic Analysis

**Figure 2:**
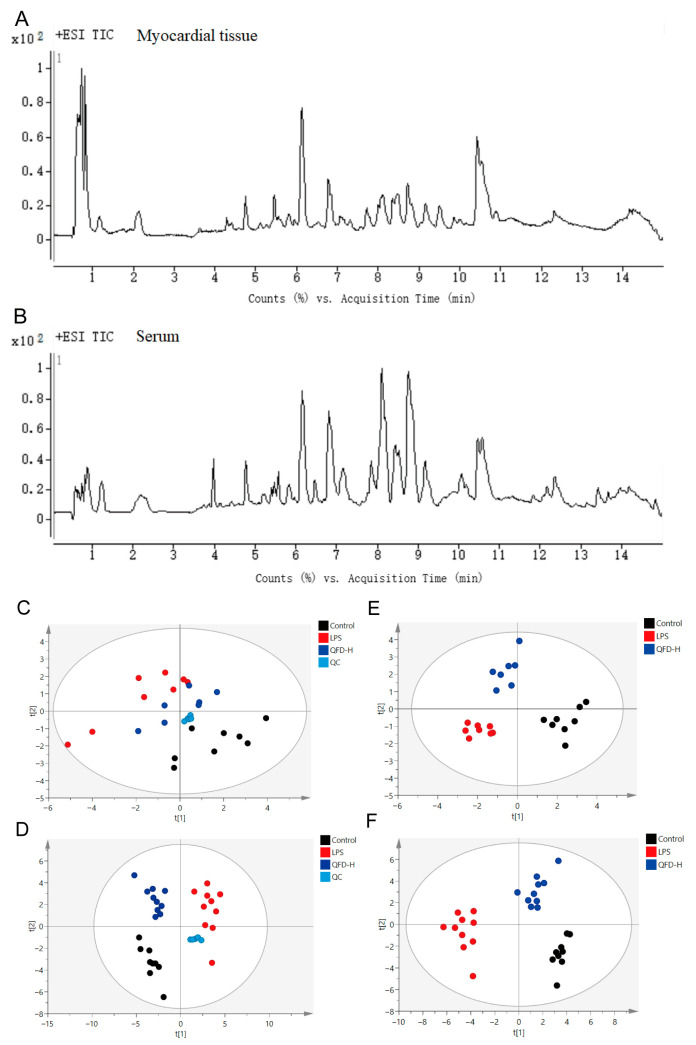
Metabolomic analysis of myocardial and serum samples. Typical total ion current (TIC) chromatogram (**A**), PCA score plots (**C**), and PLS-DA score plots (**E**) of myocardial samples. TIC chromatogram (**B**), PCA score plots (**D**), and PLS-DA score plots (**F**) of serum samples.

Typical total ion current (TIC) chromatograms are presented in Figure 2A,B. The repeatability of the analytical strategy was assessed by calculating the relative standard deviation (RSD) of the intensity of all peaks in the QC samples, and 91.5% ion features in the serum QC sample and 90.0% in the myocardial QC sample were less than 30% after peak alignment, filtering, and normalization, suggesting good repeatability of the analytical strategy. Additionally, PCA score plots of the real samples and QC sample clearly showed that QCs clustered tightly together (Figure 2C,D), forcefully confirming the stability and reliability of the analytical strategy. PLS-DA score plots showed an obvious separation among the control, LPS, and QFD-H groups, with the QFD-H group being close to the control group (Figure 2E,F), which was consistent with the PCA score plots. These results suggest that LPS disrupted metabolism in the SIC mice and that QFD-H could reverse the metabolic disorders.

### 2.4. Identification of Differential Metabolites

OPLS-DA score plots of the myocardial and serum samples showed that the control group was distinctly separated from the LPS group (Figure S2A,C). OPLS-DA models were further validated by 200 random permutation tests. The R^2^cum and Q^2^cum values after permutation were below the raw values (Figure S2B,D), indicating that the models were not overfitting. Differential metabolites were then selected based on the criteria of VIP > 1.0 and *p* < 0.05. In the myocardial samples, 23 metabolites were recognized as biomarkers of SIC, 18 of which were evidently reversed by QFD-H (Table S2). In the serum samples, 16 metabolites were identified as biomarkers of SIC, 9 of which were evidently reversed by QFD-H (Table S3).

### 2.5. Metabolic Pathway Analysis

**Figure 3:**
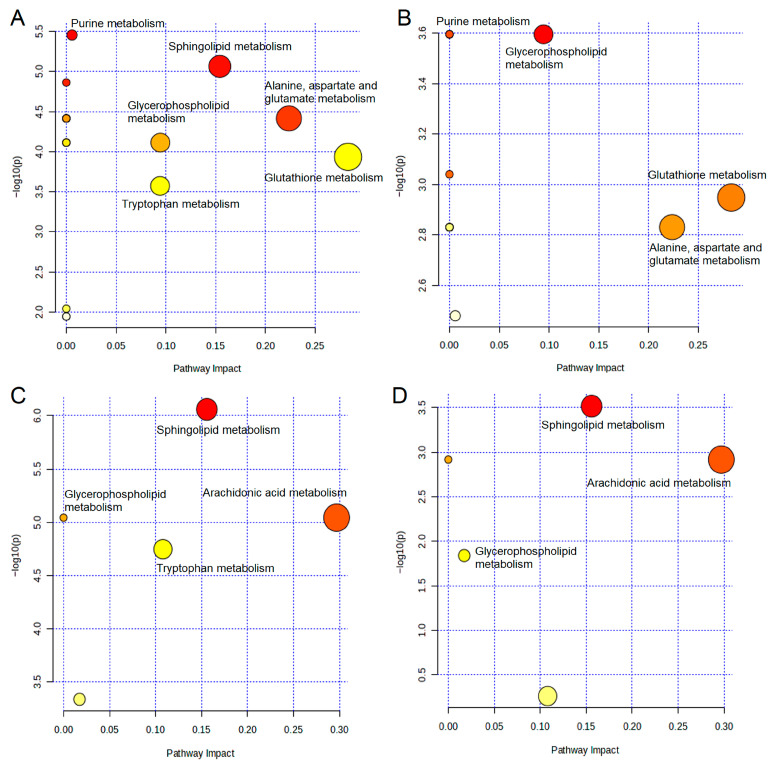
Metabolic pathway enrichment analysis of the significantly changed metabolites in myocardial samples of LPS-induced mice (**A**) and QFD-treated mice (**B**) and in serum samples of LPS-induced mice (**C**) and QFD-treated mice (**D**). The larger the circle, the greater the impact on this pathway, and the redder the circle, the higher the credibility.

**Figure 4:**
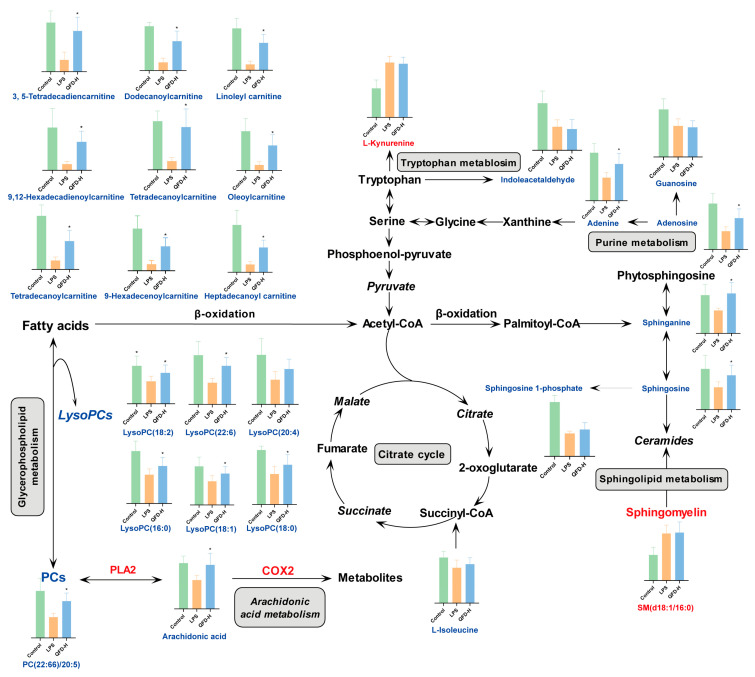
KEGG database-based metabolic network of LPS-induced cardiomyopathy and QFD modulation. Significantly increased and decreased metabolites in LPS group compared to in control group are marked in red and blue, respectively. Data are expressed as mean ± SD. * *p* < 0.05, compared with LPS group.

To investigate potential LPS-perturbed and QFD-modulated metabolic pathways, MetaboAnalyst 6.0 was applied to analyze the metabolites that notably changed and were reversed in the LPS (vs. control) and QFD-H (vs. LPS) groups. In the myocardial samples, six LPS-perturbed metabolic pathways were identified, namely, glutathione metabolism; alanine, aspartate, and glutamate metabolism; sphingolipid metabolism; glycerophospholipid metabolism; tryptophan metabolism; and purine metabolism (Figure 3A). The metabolic pathways modulated by QFD were glutathione metabolism; alanine, aspartate, and glutamate metabolism; glycerophospholipid metabolism; and purine metabolism (Figure 3B). In the serum samples, four LPS-perturbed metabolic pathways were identified, namely, arachidonic acid metabolism, sphingolipid metabolism, tryptophan metabolism, and glycerophospholipid metabolism (Figure 3C). The metabolic pathways modulated by QFD were arachidonic acid metabolism, sphingolipid metabolism, and glycerophospholipid metabolism (Figure 3D). A total metabolic network diagram is shown in Figure 4.

### 2.6. Network Pharmacology-Based Mechanism Analysis of QFD Against SIC

**Figure 5:**
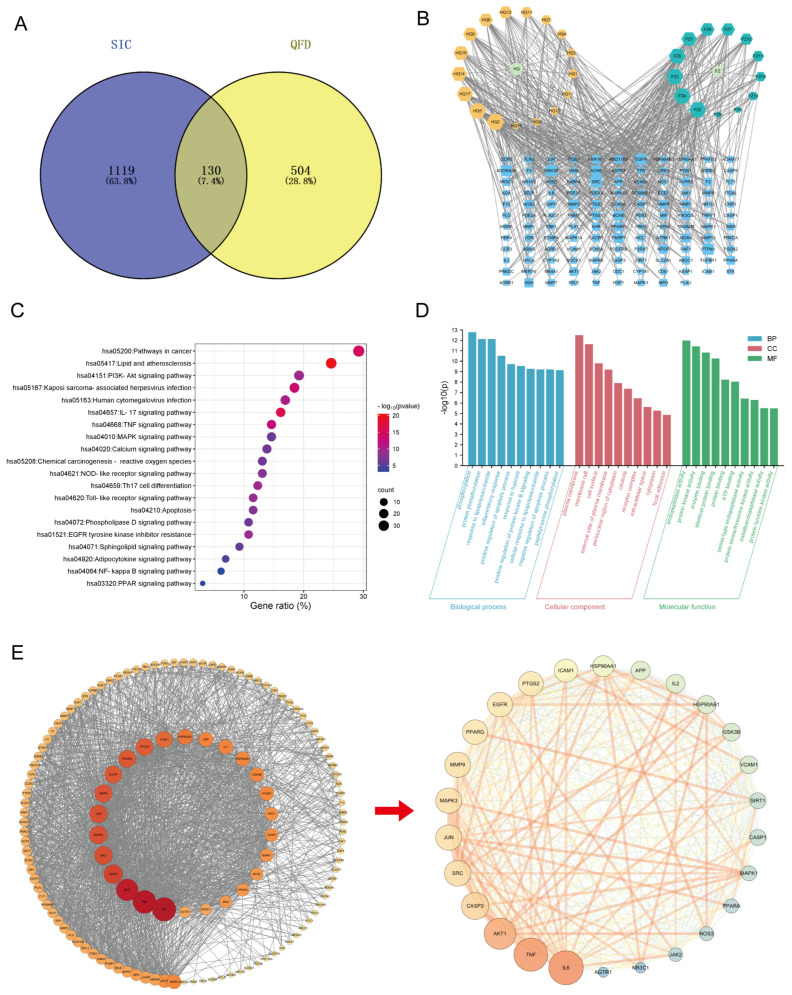
Network pharmacology-based mechanism analysis of QFD against SIC. (**A**) Wayne diagram. (**B**) Component–target network diagram of QFD against SIC. Components and genes are shown in different sizes according to their degree values. HQ 1-17 and FZ 1-13 correspond to the compositional information in Tables S4 and S5, respectively. Abbreviations: HQ, Huangqi; FZ, Fuzi. (**C**) KEGG pathway bubble map. (**D**) Bar chart of GO enrichment analysis for the BP-CC-MF triad. (**E**) Core genes screened from the PPI network. On the left is the visualized PPI network mapped with Cytoscape. The larger the degree value of the gene, the larger the shape and the redder the color. On the right is the network of the 26 core genes screened using Cytoscape. The larger the shape and the more orange the color, the more significant their role in the whole network. The thicker the connecting line between the genes, the more significant the interaction between the genes.

Using multiple databases, 17 and 13 active ingredients were screened in Huangqi (Table S4) and Fuzi (Table S5), respectively, and a total of 634 target genes for QFD and 1249 target genes for SIC were obtained. A Wayne diagram (Figure 5A) showed that there were 130 intersecting genes between QFD and SIC. Combining the above results, a component–target network diagram of QFD against SIC (Figure 5B) was plotted using Cytoscape. Different components and genes are presented in different sizes according to their degree values, which makes it clear to observe the degree of influence of each node.

In the KEGG enrichment analysis, 154 KEGG pathways were identified with FDR (corrected *p*-value) < 0.05 as a filtering condition. After sorting the pathways in ascending order of the FDR, the top 20 pathways were selected, and a KEGG pathway bubble map was drawn (Figure 5C). The intersecting genes were evidently involved in lipid and atherosclerosis, IL-17, TNF, toll-like receptor, NOD-like receptor, apoptosis, calcium, chemical carcinogenesis-reactive oxygen species, phospholipase D, sphingolipid, NF-κB, and PPAR signaling pathways. Taken together, these pathways with significant effects were primarily associated with inflammation, apoptosis, oxidative stress, and energy regulation.

The GO enrichment analysis encompassed three dimensions: biological process (BP), cellular component (CC), and molecular function (MF). With FDR < 0.05 as the filtering condition, all data were arranged in ascending order of the FDR. The top 10 nodes of each of the three dimensions were selected, and a bar chart of the GO enrichment analysis of the BP-CC-MF triad was plotted (Figure 5D). The intersecting genes were significantly involved in phosphorylation, protein phosphorylation, the response to lipopolysaccharide, the inflammatory response, and other biological processes. Moreover, they were mainly localized in the plasma membrane, membrane raft, cell surface, etc., and they had endopeptidase activity, protein kinase activity, enzyme binding, identical protein binding, protein binding, and other molecular functions.

A PPI network of the intersecting genes between QFD and SIC was constructed, as shown in Figure S3, and it was visualized using Cytoscape in accordance with the gene degree values. To reveal the core targets and key mechanism of QFD against SIC, the core genes were further screened (Figure 5E), and 26 core genes were arranged in descending order according to the degree value in Table S6, namely, IL-6, TNF, AKT1, CASP3, SRC, MAPK3, JUN, MMP9, EGFR, PPARg, PTGS2, ICAM1, HSP90AA1, APP, IL2, HSP90AB1, GSK3B, VCAM1, SIRT1, CASP1, MAPK1, NOS3, PPARα, JAK2, NR3C1, and AGTR1. Observably, IL-6 and TNF, two pro-inflammatory cytokines, played crucial roles in the whole network.

### 2.7. QFD Significantly Mitigated Inflammation in SIC Mice and RAW 264.7 Cells

**Figure 6:**
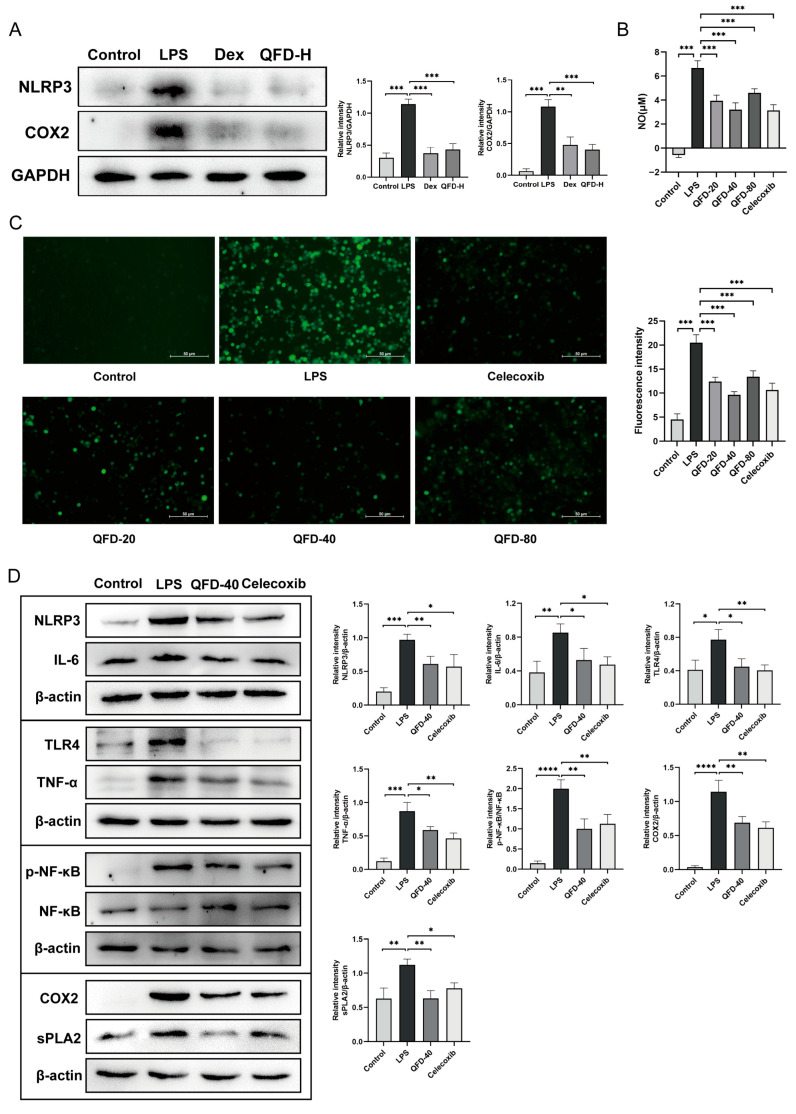
The anti-inflammatory effect of QFD on SIC mice and RAW 264.7 cells. (**A**) The expression levels of NLRP3 and COX2 in the myocardium of SIC mice (n = 3). (**B**) The content of NO in RAW 264.7 cells (n = 5). (**C**) ROS fluorescence images of RAW264.7 cells (scale bar = 50 μm, original magnification 100×) and the ratio of ROS fluorescence intensity (n = 6). (**D**) The expression levels of NLRP3, IL-6, TLR4, TNF-α, p-NF-κB, NF-κB, COX2, and sPLA2 in RAW 264.7 cells (n = 3). RAW 264.7 cells were pretreated with different concentrations of QFD (20, 40, and 80 μg/mL) and 1 μM celecoxib as a positive drug group for 5–6 h, and then they were treated with LPS (1 μg/mL) for 24 h to establish an inflammatory cell model. The NO, ROS, and protein levels of the cells were further measured. Data are expressed as mean ± SD. * *p* < 0.05, ** *p* < 0.01, *** *p* < 0.001, **** *p* < 0.0001, ns represents no significant difference.

Given the many inflammatory pathways and targets identified by metabolomics and network pharmacology, we further verified the ameliorative effect of QFD on inflammation. The expression levels of NLRP3 and COX2 were measured in the myocardium of the SIC mice, and the results showed that two proteins significantly increased in the LPS group and significantly decreased after QFD and Dex treatment (Figure 6A). Macrophage-mediated inflammation has been shown to cause SIC [18,19], so LPS-induced RAW 264.7 cells were used to further investigate the potential anti-inflammatory mechanism of QFD during SIC. Upon exposure to LPS, the NO and ROS production of RAW 264.7 cells notably increased, which was significantly reverted by QFD (20, 40, and 80 μg/mL) (Figure 6B,C). Due to having the most pronounced effect, QFD-40 was chosen as the treatment group for subsequent Western blot analysis. As shown in Figure 6D, we measured the protein levels of TLR4 and p-NF-κB p65, including their downstream NLRP3, IL-6, and TNF-α. Moreover, the protein levels of sPLA2 and COX2 were also determined. QFD treatment significantly decreased the expression of the above proteins. These results suggest that QFD could regulate the TLR4/NF-κB/NLRP3 inflammatory pathway, glycerophospholipid metabolism, and arachidonic acid metabolism, thereby exerting an anti-inflammatory effect in SIC.

### 2.8. QFD Alleviated SIC by Modulating Impaired FAO Through Activation of PPARα/CPT Pathway

**Figure 7:**
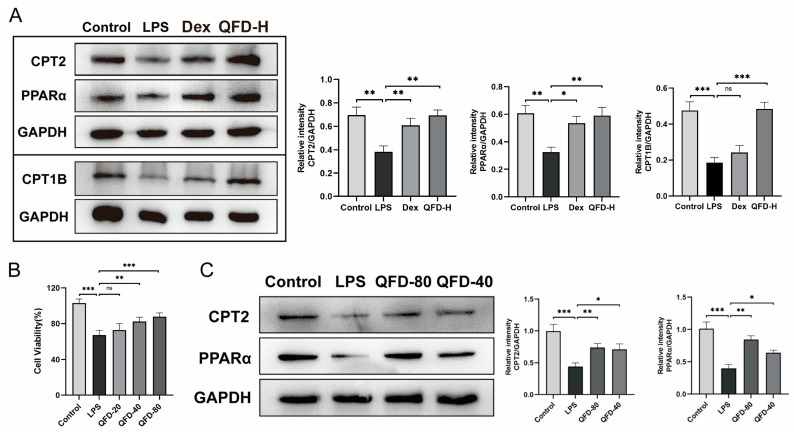
Alleviated effect of QFD on PPARα/CPT pathway in myocardium of SIC mice and H9c2 cells. (**A**) Expression levels of PPARα, CPT2, and CPT1B in myocardium of SIC mice (n = 3). (**B**) Cell viability (%) of H9c2 cells treated with various concentrations of QFD (n = 5). (**C**) Expression levels of PPARα and CPT2 in H9c2 cells (n = 3). H9c2 cells were pretreated with QFD (20, 40, and 80 μg/mL) for 5-6 h and were subsequently stimulated with supernatant collected from RAW 264.7 cells for 24 h to generate an SIC cell model. Cell viability and protein levels of H9c2 cells were then determined. Data are expressed as mean ± SD. * *p* < 0.05, ** *p* < 0.01, *** *p* < 0.001, ns represents no significant difference.

Due to the notable changes in the content of acyl-carnitines in metabolites, it was speculated that fatty acid oxidation (FAO) was affected during SIC. In addition, network pharmacology suggested that the PPAR signaling pathway and PPARα play an important role in QFD against SIC. Therefore, the expression levels of three key proteins involved in FAO, namely, PPARα [20] and its downstream CPT1B and CPT2 [21], were determined. The levels of the three proteins were evidently higher in the heart tissues of the SIC mice in the QFD and Dex groups than in those in the LPS group (Figure 7A). Based on the results of a CCK-8 assay (Figure 7B), two concentrations of QFD-40 and QFD-80 were selected for further Western blot analysis, and QFD also evidently upregulated the expression of PPARα and CPT2 in H9c2 rat cardiomyocyte cells (Figure 7C).

### 2.9. Molecular Docking Analysis and Efficacy Analysis of the Active Ingredients in QFD

**Figure 8:**
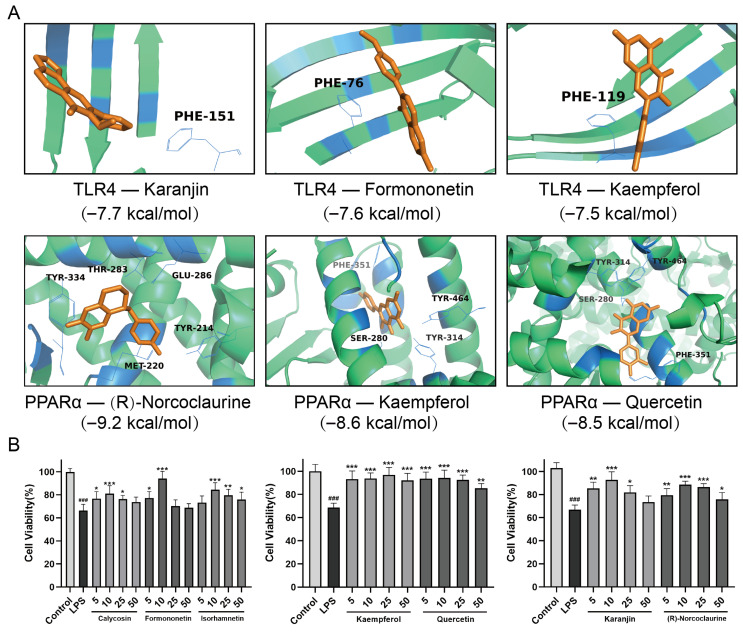
Molecular docking analysis and efficacy analysis of the active ingredients in QFD. (**A**) Binding patterns of TLR4 and PPARα with some components with strong interactions. (**B**) Cell viability (%) of H9c2 cells treated with various active ingredients of QFD (n = 6). H9c2 cells were pretreated with calycosin, formononetin, isorhamnetin, kaempferol, quercetin, karanjin, and (R)-norcoclaurine (5, 10, 25, and 50 μM) for 5–6 h and were subsequently stimulated with supernatant collected from RAW 264.7 cells for 24 h to generate an SIC cell model. Cell viability and protein levels of H9c2 cells were then determined. Data are expressed as mean ± SD. ^###^ *p* < 0.001, compared with the control group; * *p* < 0.05, ** *p* < 0.01, *** *p* < 0.001, compared with the LPS group.

Based on the results of the ingredient identification of QFD and network pharmacology, nine components were docked with two upstream targets, PPARα and TLR4, to further validate the effects of QFD on both the TLR4/NF-κB/NLRP3 and PPARα/CPT pathways. It is generally believed that docking binding energies below −5.0 kcal/mol indicate good binding activity, while energies below −7.0 kcal/mol indicate strong binding activity [22]. Table S7 displays the docking scores, and most active ingredients had a docking binding energy of less than −7.0 kcal/mol with these two targets, indicating a high affinity. MM/GBSA was used to determine the binding free energy of the docked compounds. The more negative the binding free energy value, the greater the stability of the complex. Table S8 shows the MM-GBSA results, which were all negative, confirming stable binding to the protein. Figure 8A shows the binding patterns of TLR4 and PPARα with some components with strong interactions; for example, karanjin bound to TLR4 through PHE-151, and (R)-norcoclaurine bound to PPARα through five amino acids: TYR-334, THR-283, GLU-286, TYR-214, and MET-220. Additionally, we verified the ameliorative effect of seven active ingredients on SIC by measuring the viability of H9c2 cardiomyocytes. As shown in Figure 8B, compared with the LPS group, these seven active ingredients all led to a significant increase in the cell survival rate, especially kaempferol and quercetin, which showed good protective effects at 5 μM.

## 3. Discussion

Sepsis-induced cardiomyopathy (SIC) is a cardiac dysfunction that occurs during sepsis with symptoms such as decreased left ventricular systolic function and reduced ejection fraction [7], and it is one of the most serious complications of sepsis [23,24,25]. Numerous studies have shown that the pathogenesis of SIC is mainly based on excessive inflammation, impaired myocardial energy metabolism, apoptosis, oxidative stress, and calcium overload [26,27]. Qifu decoction (QFD) is an ancient TCM compound used to treat cardiovascular diseases and is composed of Huangqi and Fuzi in a mass ratio of 2:1. Among them, Fuzi is rich in various alkaloids and has cardiotonic effects [28]. However, it has potential cardiotoxicity, with a relatively narrow margin of safety. Cai et al. emphasized the dose-dependent cardiotoxicity of Fuzi [29]. The Chinese Pharmacopoeia (ChP) stipulates that the maximum daily dose of Fuzi for an adult is 15 g. Calculated based on an adult body weight of 70 kg, the daily dose for mice is approximately 2.5 g/kg [15]. To prevent the potential cardiac toxicity of QFD, we set the dose of QFD to 3 and 6 crude herbs/kg body weight, and the doses of Fuzi were 1 and 2 g/kg in theory, respectively, which were lower than the maximum dose specified in the ChP. Additionally, our previous research also demonstrated that the current dosage of QFD does not cause cardiac toxicity and is within a safe range [30], which can provide a reference for the clinical treatment of QFD.

**Figure 9:**
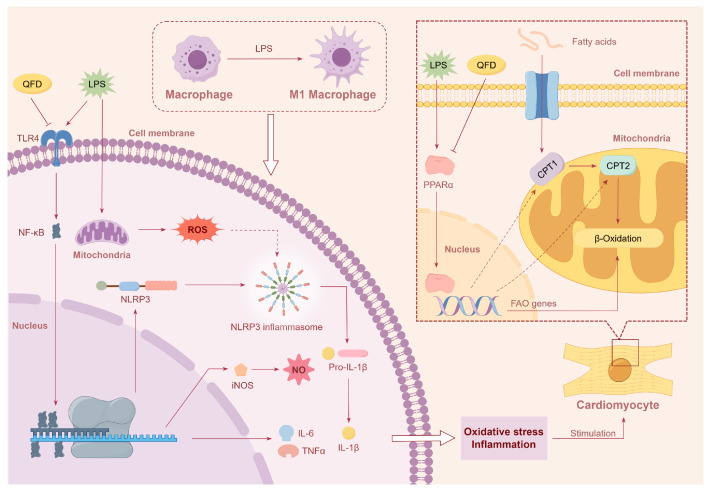
Overall mechanism diagram of QFD against SIC by suppressing TLR4/NF-κB/NLRP3 inflammatory pathway and modulating impaired FAO through activation of PPARα/CPT pathway. This figure was created by Figdraw (https://www.figdraw.com/).

In this work, using electrocardiography, histopathological examination, and biochemical indicator determination, it was found that QFD could effectively ameliorate cardiac dysfunction and myocardial injury in LPS-induced SIC mice and reduce the levels of serum inflammatory factors, suggesting that QFD has a certain therapeutic effect on SIC. Metabolomics and network pharmacology were combined to further reveal the potential mechanisms of QFD against SIC, and the main focus was on the modulation of QFD on inflammation and myocardial energy metabolic homeostasis. Our results demonstrate that QFD alleviated SIC by suppressing the TLR4/NF-κB/NLRP3 inflammatory pathway and modulating impaired FAO through activation of the PPARα/CPT pathway. An overall mechanism diagram of QFD in alleviating LPS-induced myocardial dysfunction is shown in Figure 9.

### 3.1. Inflammatory Pathway

Among the pathways obtained from the KEGG pathway enrichment analysis, most were found to be inflammation-associated pathways, such as the IL-17, TNF, toll-like receptor, NOD-like receptor, NF-κB signaling pathways. The GO analysis also indicated that intersecting genes were primarily involved in the response to lipopolysaccharide and the inflammatory response. Among the core genes screened, IL-6 and TNF, two pro-inflammatory cytokines, had crucial effects on the overall network, and they were involved in multiple pathways, as described above. Based on these results, it was hypothesized that the inhibitory effect of QFD on SIC might occur mainly through the inhibition of the inflammatory response. Toll-like receptor 4 (TLR4) is a pattern recognition receptor that directly binds to bacterial LPS and plays a crucial role in the innate immune response. A large number of studies have indicated that TLR4 can contribute to SIC [31]. The activation of TLR4 initiates key downstream signaling molecules, such as NF-κB, which is critical in the pathogenesis of SIC [32]. After activation, NF-κB enters the nucleus and undergoes phosphorylation, further upregulating the levels of downstream genes such as IL6, TNF-α [33], and NLRP3 [34]. The NLRP3 inflammasome is composed of NLRP3, ASC, and pro-caspase-1, regulating the activation of caspase-1 and following the release of IL-1β and IL-18 from innate immune cells during infection or damage. The TLR4/NF-κB pathway and its downstream NLRP3 inflammasome have been demonstrated to be intricately linked with SIC [35,36,37]. Therefore, we further validated the moderation of QFD on the TLR4/NF-κB/NLRP3 pathway. As predicted by network pharmacology, TLR4 had strong docking activity with the active ingredients of QFD. There were multiple flavonoids in these active ingredients. Many studies have confirmed that flavonoids such as formononetin, calycosin, quercetin, and kaempferol have the ability to alleviate oxidative stress and inflammation, demonstrating good cardioprotective effects [38,39,40]. This indicates that flavonoids play an important role in the process of QFD regulating the inflammatory pathway. The protein levels of TLR4 and p-NF-κB p65 and their downstream NLRP3, IL-6, and TNF-α in LPS-induced RAW 264.7 cells were significantly downregulated by QFD, indicating that QFD potentially relieved SIC by inhibiting the TLR4/NF-κB/NLRP3 pathway.

Metabolically, it was a significant finding that the levels of PC(22:6)/20:5), LysoPCs (including LysoPC(16:0), LysoPC(18:0), LysoPC(18:1), LysoPC(18:2), LysoPC(20:0), and LysoPC(22:6)), and arachidonic acid (AA) in the LPS group evidently decreased, which indicated that glycerophospholipid metabolism and arachidonic acid metabolism were impeded during SIC. Under the catalysis of phospholipase A2 (PLA2), phosphatidylcholines (PCs) are hydrolyzed to produce LysoPC and AA. AA can be further metabolized by cyclooxygenases (COXs) to form bioactive prostaglandins [41]. Additionally, PTGS2 (COX2) was found to be a core target of QFD against SIC in network pharmacology. Several studies have confirmed that the levels of PLA2 and COX2 are associated with cardiac dysfunction and myocardial inflammation [42,43]. As expected, Western blot revealed that the levels of sPLA2 and COX2 were upregulated in the LPS-induced SIC mice and RAW 264.7 cells, which were significantly downregulated by QFD. The above findings that we obtained suggest that the modulation of the PLA2/COX2 pathway by QFD seems to be a critical point in inhibiting SIC.

### 3.2. Metabolic Homeostasis

The decrease in acyl-carnitines indicated that fatty acid oxidation (FAO) was unbalanced in the LPS-induced myocardium. FAO is vital for energy provision in the heart, and any abnormalities in this process can lead to myocardial damage [44]. A significant decrease in FAO is a feature of cardiovascular diseases, including SIC, which may result in disturbances in myocardial energy metabolism and exacerbate energy expenditure, contributing to myocardial cell death [45]. Peroxisome proliferator-activated receptor α (PPARα), a well-known nuclear receptor, influences fatty acid homeostasis through the regulation of fatty acid transport, FAO, and lipid droplet formation [46]. The absence of PPARα results in a shift in cardiac metabolism from FAO to glucose oxidation and lactic acid production, which is not enough to sustain ATP production and cardiac function. The absence of PPARα causes a reduction in cardiac performance and FAO in sepsis [47]. The network pharmacology results suggested that the PPAR signaling pathway and two core genes, PPARg and PPARα, played significant roles in QFD against SIC. A recent study demonstrated that the gene levels of PPARα in the heart of SIC mice were most significantly decreased in the PPAR family (PPARα, PPARd, and PPARg) and that PPARα might have an important effect in LPS-induced cardiac dysfunction [48].

The expression of multiple genes involved in FAO can be regulated by PPARα [20], including carnitine palmitoyltransferase (CPT) [49,50], a key rate-limiting system in FAO. The activation of PPARα enhances CPT expression [49]. CPT contains CPT1 and CPT2, two main subtypes that are situated separately in the outer and inner mitochondrial membranes, and it is mainly responsible for the transport of fatty acids (FAs) in FAO [51]. During FAO, FAs and carnitines enter the cell through transmembrane proteins. After the conversion of FAs to acyl coenzyme A (CoA), carnitines are converted to acyl-carnitines by CPT1 and enter the mitochondria. Subsequently, acyl-carnitines are converted back to carnitine and acyl CoA by CPT2 [52], which further initiates β-oxidation [53]. There are three subtypes of CPT1, namely, CPT1A, CPT1B, and CPT1C, with CPT1B predominantly found in the heart [54]. In our study, the protein levels of PPARα and its downstream targets CPT1B and CPT2 in the myocardium of SIC mice were evidently reduced after LPS induction. This might be due to the inhibition of PPARα activity by LPS, which reduced CPT1B and CPT2 gene expression. Moreover, several studies have shown that reactive oxygen species (ROS), nitric oxide (NO), and peroxynitrite produced in the heart during sepsis inhibit CPT1 activity [55,56]. The reduced CPT1B expression level and activity impeded carnitine acylation, which might be the main reason for the decreased levels of acylcarnitines in metabolites.

Studies have demonstrated that, in the early stages of sepsis, interferon-γ and LPS can induce macrophage M1-like polarization, thereby promoting the production of inflammatory factors [57]. This damages cardiomyocytes and triggers cardiac dysfunction [58]. During sepsis, cardiomyocyte damage may be attributed to activation of M1-like macrophages [59,60], and the levels of inflammatory factors reach their peak around 12–24 h [61]. LPS stimulation alone did not significantly affect the induction of inflammatory damage in H9c2 cells [62]. Therefore, we co-cultured H9c2 cells with culture supernatants from LPS-stimulated RAW 264.7 cells to simulate the state of myocardial cell damage caused by sepsis. We finally found a significant decrease in cell viability and a decrease in the protein levels of PPARα and CPT2. Based on these results, we speculated that LPS induced the M1 polarization of macrophages, which increased NO and ROS production and activated the TLR4/NF-κB/NLRP3 pathway, thereby producing pro-inflammatory factors such as IL-6 and TNF-α, which, in turn, led to cardiomyocyte damage and FAO impairment (Figure 9). QFD significantly restored the levels of PPARα, CPT1B, and CPT2, suggesting that it can regulate FAO by activating the PPARα/CPT pathway, thereby maintaining myocardial energy metabolic homeostasis, which may be another key mechanism of QFD against SIC.

The results of molecular docking show that a compound may have multiple targets, which might produce potential off-target effects. The off-target effects of drugs are closely related to their safety and efficacy. On the one hand, off-target effects can directly impact therapeutic efficacy and produce side effects and toxicity. On the other hand, they can also bring unexpected benefits. For example, a drug may not only act on its main target but also have beneficial effects on other related molecules, thereby enhancing the overall therapeutic effect. Therefore, it is necessary to conduct in-depth research on the targets of the screened compounds, potential off-target effects, and the results caused by off-target effects.

## 4. Materials and Methods

### 4.1. Reagents

LPS was purchased from Sigma-Aldrich (St. Louis, MO, USA). Dexamethasone was purchased from Henan Runhong Pharmaceutical Co., Ltd. (Zhengzhou, China). Dulbecco’s modified eagle medium, fatal bovine serum, and a cell counting kit-8 assay were purchased from Keygen Biotech Co., Ltd. (Nanjing, China). ELISA kits for CK-MB, IL-1β, and TNF-α were purchased from AiFang Biological Co., Ltd. (Changsha, China). An LDH assay kit was purchased from Nanjing Jiancheng Bioengineering institute Co., Ltd. (Nanjing, China). The antibodies used for Western blot in this study were as follows: anti-NLRP3 (1:1000, ab263899, Abcam, Waltham, MA, USA), anti-TLR4 (1:1000, 14358, Cell Signaling Technology, Danvers, MA, USA), anti-COX2 (1:1000, ab62331, Abcam, Waltham, MA, USA), anti-TNF-α (1:1000, 17590-1-AP, Proteintech, Rosemont, IL, USA), anti-sPLA2 (1:1000, M02259-1, BOSTER, Hangzhou, China), anti-IL-6 (1:1000, DF6087, Affinity Biosciences, Cincinnati, OH, USA), anti-NF-κB p65 (1:1000, 8242, Cell Signaling Technology, Danvers, MA, USA), anti-p-NF-κB p65 (1:1000, 3033, Cell Signaling Technology, Danvers, MA, USA), anti-β-actin (1:500, AP0060, Bioworld, Visalia, CA, USA), anti-CPT2 (1:1000, 26555-1-AP, Proteintech, Rosemont, IL, USA), anti-CPT1B (1:500, 22170-1-AP, Proteintech, Rosemont, IL, USA), anti-PPARα (1:1000, ab314112, Abcam, Waltham, MA, USA), anti-GAPDH (1:500, AP0063, Bioworld, Visalia, CA, USA), and goat anti-rabbit IgG (H+L) HRP (1:10,000, BS13278, Bioworld, Visalia, CA, USA). RIPA lysate, a BCA assay kit, a reactive oxygen species assay kit, and an NO assay kit were purchased from Beyotime Biotechnology (Shanghai, China). 1 × PBS, bovine serum albumin, anti-CD68 antibody, and 3,3′-diaminobenzidine chromogenic solution were purchased from Servicebio Co., Ltd. (Wuhan, China). Songorine (CAS: 509-24-0), astragaloside IV (CAS: 83207-58-3), formononetin (CAS: 485-72-3), calycosin (CAS: 20575-57-9), isorhamnetin (CAS: 480-19-3), karanjin (CAS: 521-88-0), and (R)-norcoclaurine (CAS: 5843-65-2) were purchased from Desite Co., Ltd. (Chengdu, China). Quercetin (CAS: 117-39-5) and kaempferol (CAS: 520-18-3) were purchased from aladdin Co., Ltd. (Shanghai, China).

### 4.2. Preparation and Ingredient Identification of QFD

Dried herbal pieces, including 30 g of Huangqi (HQ20230223, Gansu province) and 15 g of Fuzi (FZ20230219, Sichuan province), were soaked together in 10-fold deionized water (*v*/*w*) for 1 h, followed by two extractions using boiling water for 2 h and 1 h. Thereafter, the extract was filtered, combined twice, and concentrated into 0.5 g/mL (crude drug concentration) QFD. The ingredient identification and quantitative analysis of QFD were conducted by UPLC-QTOF-MS (Agilent 6545, Santa Clara, CA, USA), and specific operations are shown in the Supplementary Methods.

### 4.3. Animals

Fifty-eight male ICR mice (8–12 weeks, 24–26 g) were obtained from the Comparative Medicine Center of Yangzhou University (SCXK (Su) 2022-0009, Yangzhou, China). The mice were adaptively raised for 7 days in a routine environment (temperature 25 ± 2 °C, 12 h light/dark cycle, humidity 55 ± 10%) before the experiment was officially carried out. The whole experimental process was approved by the Animal Ethics Committee of China Pharmaceutical University.

### 4.4. LPS-Induced Sepsis Model Construction and Treatment

A lipopolysaccharide (LPS)-induced sepsis model is an accepted mouse model for studying sepsis-induced myocardiopathy [19]. Dexamethasone (Dex) was used as the positive drug [63]. The mice were randomly divided into 5 groups: control group (n = 10), LPS group (n = 12), QFD low-dosage (QFD-L) group (n = 12), QFD high-dosage (QFD-H) group (n = 12), and Dex group (n = 12). The mice in the QFD-L and QFD-H groups were individually administered intragastrically with QFD at a dosage of 3 g and 6 g crude herbs/kg body weight for 7 successive days. The dosage selection of QFD was based on our previous research [30] and complied with the regulations of the Chinese Pharmacopoeia. The Dex group mice were injected intraperitoneally continuously at a dose of 10 mg/kg for 7 days. The control and LPS groups were intragastrically treated with the same volume of physiological saline. Then, 1 h after the end of administration on the 7th day, except for the control group, the mice in the other groups were intraperitoneally injected with 10 mg/kg LPS to establish a septic model [64]; 12 h after the LPS injection, cardiac function was measured, and blood samples and heart tissues of the mice were collected.

### 4.5. Cardiac Function Measurements

After LPS treatment for 12 h, the mice were anesthetized with 1.5% isoflurane, and their cardiac function was detected using a Vevo 3100LT Imaging System (FUJIFILM VisualSonics, Toronto, ON, Canada). A two-dimensional M-mode ultrasound was selected for echocardiography. Left ventricular ejection fraction (LVEF) and left ventricular fractional shortening (LVFS) were further obtained.

### 4.6. Histopathological Analysis

Freshly collected heart tissues were fixed in 4% paraformaldehyde for 24 h and embedded in paraffin. Afterward, the heart tissues were cut into sections with a thickness of 4–5 μm and stained with hematoxylin–eosin (HE). Changes in myocardial tissue morphology were observed through an upright microscope (Leica, Wetzlar, Germany).

### 4.7. Immunohistochemistry

The paraffin-embedded heart tissues were deparaffinized, incubated in a 3% hydrogen peroxide solution in the dark for 25 min, and washed 3 times with 1×PBS. Then, 3% bovine serum albumin was added dropwise into the histochemical circle and sealed for 30 min. The sections were incubated with anti-CD68 primary antibody at 4 °C overnight, followed by incubation with goat anti-rabbit IgG (H+L) HRP for 50 min at RT after 3 washes. Afterward, 3,3′-diaminobenzidine (DAB) chromogenic solution was added dropwise, and the color development time was controlled under a microscope. The positive color was brownish-yellow, and the sections were rinsed with tap water to terminate color development. Hematoxylin was used to re-stain the nuclei of the cells, and then the sections were dehydrated, sealed, and placed under an upright microscope for observation.

### 4.8. Assessment of Biochemical Indicators and Inflammatory Factors

The serum levels of creatine kinase MB (CK-MB), lactate dehydrogenase (LDH), interleukin-1β (IL-1β), and tumor necrosis factor-α (TNF-α) of the SIC mice were assessed using ELISA kits according to the manufacturers’ instructions.

### 4.9. Sample Preparation for Metabolomic Analysis

Serum and heart tissues from the SIC mice were subjected to metabolomic analysis. For the serum samples, a 4-fold volume of pre-cooled methanol containing the internal standard (2 μg/mL L-2 chlorophenylalanine) was added to the serum. The mixture was vigorously vortex for 1 min, followed by 14,000 rpm centrifugation for 15 min at 4 °C. Finally, 100 µL supernatant was obtained for further metabolomic analysis. For the heart tissues, 20-fold pre-cooled 80% methanol containing 2 μg/mL L-2 chlorophenylalanine (*v*/*w*) was added to 20–30 mg heart tissues. The mixture was fully ground with a freeze grinder (JXFSTPRP-CL-24, Shanghai, China), followed by 14,000 rpm centrifugation for 15 min at 4 °C. The supernatant was collected for metabolomic analysis. Aliquots of each sample were pooled as a quality control (QC) sample to monitor the repeatability of the metabolomic workflows.

### 4.10. Metabolomic Analysis

The serum and heart metabolites were separated through an Agilent 1290 Infinity II liquid chromatography system equipped with an ACQUITY UPLC^®^ BEH C18 column (Waters, Milford, MA, USA) (2.1 × 100 mm, 1.7 μm) at 40 °C and detected with an Agilent 6545 Quadrupole Time-of-Flight (Q-TOF) mass spectrometer. The mobile phase consisted of ultra-pure water–acetonitrile (95:5, *v*/*v*) containing 0.1% formic acid (A) and acetonitrile containing 0.1% formic acid (B). The elution gradient and instrument parameters were as follows: 0–2 min, 0% B; 2–5 min, 0–50% B; 5–13 min, 50–85% B; 13–14 min, 85–95% B; 14–15 min, 95% B; column oven, 40 °C; injection volume, 4 μL; autosampler temperature, 4 °C; flow rate, 0.4 mL/min. Notably, the QC sample was initially injected 5 times before starting the sequence, followed by an injection after every eight random samples. The Q-TOF-MS was operated in full-scan MS resolution mode in positive ionization mode (ESI+). The instrument parameters were programmed as follows: dry gas temperature, 350 °C; capillary voltage, 3.5 kV; spray pressure, 45 psig; drying gas velocity, 11 L/min; fragmentation voltage, 120 V; Skimmer voltage, 60 V; collision energy, 10 V, 20 V, and 40 V; mass/charge ratio collection range, 50–1000 *m*/*z*.

The chromatographic data were then converted into mzData format through MassHunter Qualitative Analysis B.10.0 software. The XCMS package was used for chromatographic peak recognition, extraction, comparison, filtering, and filling. The “80% rule” was used to screen the missing peaks, followed by normalizing the areas of all chromatographic peaks with the internal standard. SIMCA 14.1 software (Umetrics, Umea, Sweden) was employed for further multivariate data analysis. Principal component analysis (PCA) was used to examine the stability and reliability of the analytical strategy. The dispersion trend among the control, LPS, and QFD groups was displayed by partial least squares discriminant analysis (PLS-DA). The difference between the LPS group and control group and the variable importance in projection (VIP) of each metabolite were obtained from orthogonal partial least squares discriminant analysis (OPLS-DA), and VIP > 1 and *p* < 0.05 were the criteria for differential metabolites.

Further identification of differential metabolites was preliminarily conducted by contrasting their exact mass-to-charge ratio (*m*/*z*) with those in the HMDB and METLIN databases with a 10 ppm tolerance. MS/MS analysis of quasi-molecular ions was performed to narrow down the range of unknown metabolites. Metabolic pathway analysis based on the Kyoto Encyclopedia of Genes and Genomes (KEGG) was carried out by MetabAnalyst 6.0.

### 4.11. Network Pharmacology Analysis

The mechanism of QFD against SIC was analyzed and predicted using network pharmacology strategies. Firstly, the TCMSP database was used to screen the active ingredients of Huangqi and Fuzi in QFD based on the criteria of oral bioavailability (OB) ≥ 30% and drug-like properties (DL) ≥ 0.18. The PubChem database and Swiss database were employed to obtain the structural information of these compounds and predict the target genes of QFD, respectively. Two human gene databases, GeneCards and OMIM, were used to screen the corresponding target genes of SIC. Then, the target genes of QFD were imported into Venny 2.1.0 together with those of SIC, and the intersecting genes and a Wayne diagram were exported. KEGG pathway and GO enrichment analyses of the intersecting genes were further conducted with the DAVID database to reveal possible target pathways for the treatment of SIC. Additionally, the STRING database was employed to construct a PPI network of the intersecting genes, and core genes in the network were screened with Cytoscape 3.7.1 software. Using the centiscape plug-in, core genes were selected from the intersecting genes based on the criteria of closeness undir > 0.004, betweenness undir > 117.71, and degree undir > 27.2.

### 4.12. Culture and Treatment of RAW 264.7 Cells and H9c2 Cells

The RAW 264.7 macrophage cell line and H9c2 rat cardiomyocyte cell line were obtained from the cell bank of the Chinese Academy of Sciences (Shanghai, China) and Nanjing Keygen Biotech (KGG1103-1, Nanjing, China), respectively. The cells were cultivated in Dulbecco’s modified eagle medium containing 1% penicillin–streptomycin and 10% FBS (Gibco, Auckland, New Zealand) at 37 °C in a 5% CO_2_ incubator. The RAW 264.7 cells were seeded at a density of 5 × 10^4^ cells/well into 48-well plates and pretreated with QFD (20, 40, and 80 μg/mL) and 1 μM celecoxib as a positive drug group for 5–6 h, followed by 1 μg/mL LPS for 24 h to establish an inflammatory cell model. The culture supernatant was collected for nitric oxide (NO) measurement with an NO assay kit, and the reactive oxygen species (ROS) content in the RAW 264.7 cells was determined with an ROS assay kit.

For the H9c2 rat cardiomyocyte cells, about 6 × 10^3^ cells/well were seeded into 96-well plates and pretreated with QFD (20, 40, and 80 μg/mL) and the active ingredients of QFD (5, 10, 25, and 50 μM) for 5–6 h. Subsequently, the cells were stimulated with the supernatant collected from the RAW 264.7 cells for 24 h to generate an SIC cell model [65]. Cell viability was then determined by a cell counting kit-8 (CCK-8) assay.

### 4.13. Molecular Docking

The 2D structures of the active ingredients of Astragali Radix and Aconiti Lateralis Radix Praeparaia were retrieved from the PubChem database. Schrodinger’s LigPrep module was employed to generate ligands’ 3D conformers. The 3D crystal structures of PPARα (PDB ID:3ET1) and TLR4 (PDB ID:3FXI) were obtained from the RCSB Protein Data Bank and subjected to protein processing using Schrodinger Maestro 11.5 ’s “Protein Preparation Wizard module” to hydrogenate and remove water. The protein structure was further processed using the “Receptor Grid Generation” module to construct a grid encompassing the active region delineated by the ligand. The grid box for docking was centered at the centroid of the active site, with the active site boundary defined by a 20 Å radius around the ligand present in the crystal structure. This configuration ensured comprehensive coverage of the ligand-binding pocket and surrounding interactive residues relevant to the docking analysis. Schrödinger’s 2018 Glide tool was used in SP mode for molecular docking. The binding free energy of the ligand–protein complex was calculated and analyzed using the “Prime-MMGBSA” module in Schrodinger. The OPLS3 force field and the variable dielectric generalized Born 2.0 (VSGB) solvation model were employed to determine the binding free energy (ΔGbind) of the complex. Finally, Pymol 3.1 software was used to visualize the docking results.

### 4.14. Western Blot Analysis

Western blot was used to demonstrate the expression levels of the target protein in the heart tissues and cells. The total protein was extracted using RIPA lysate and quantified using a BCA protein assay kit. The proteins were separated by SDS-PAGE and transferred onto PVDF membranes. Then, the PVDF membranes were sealed in 5% nonfat dry milk in TBST solution for 2 h at RT and incubated at 4 °C overnight with the following primary antibodies: CPT1B, CPT2, PPARα, NF-κB p65, NLRP3, TLR4, p-NF-κB p65, COX2, sPLA2, IL-6, TNF-α, β-actin, and GAPDH. After that, the membranes were incubated for 1 h at RT with the corresponding goat anti-rabbit IgG (H+L) HRP secondary antibody. An automatic chemiluminescence image analysis system (Tanon, Shanghai, China) was used for detection, and each band’s gray value was measured by ImageJ 1.8.0. β-actin and GAPDH served as internal controls for normalization.

### 4.15. Statistical Analysis

All data are displayed as mean ± standard deviation (SD) and were analyzed using GraphPad Prism 9.0 software. One-way analysis of variance (ANOVA), followed by Tukey’s multiple comparison test, was used to compare multiple groups. *p*-value < 0.05 was considered statistically significant.

## 5. Conclusions

In the present study, we illustrated the cardioprotective effects of QFD on LPS-induced SIC mice through electrocardiography, histopathological examination, and biochemical indicator determination. QFD considerably enhanced cardiac function; attenuated myocardial injury; and reversed the abnormal secretion of LDH, CK-MB, IL-1β, and TNF-α. Metabolomic analysis showed that QFD could regulate seven metabolic pathways, namely, glutathione metabolism; alanine, aspartate, and glutamate metabolism; arachidonic acid metabolism; glycerophospholipid metabolism; purine metabolism; sphingolipid metabolism; and fatty acid metabolism. Network pharmacology suggested that the anti-SIC effect of QFD may be mediated by the TNF, toll-like receptor, NOD-like receptor, NF-κB, and PPAR signaling pathways. Additionally, 26 core targets were obtained. Molecular docking revealed that active ingredients such as formononetin, kaempferol, quercetin, and (R)-norcoclaurine in QFD could affect the activity of PPARα and TLR4. Further validation was carried out in in vitro and in vivo models, and the expression levels of TLR4, NLRP3, NF-κB, TNF-α, IL-6, COX2, sPLA2, PPARα, CPT1B, and CPT2 were significantly reversed by QFD. It was finally found that QFD might alleviate SIC by suppressing the TLR4/NF-κB/NLRP3 inflammatory pathway and modulating impaired FAO through activation of the PPARα/CPT pathway. These findings highlight that QFD might be a potential anti-SIC drug candidate. Although the present study preliminarily elucidated the cardioprotective effects and mechanisms of QFD against SIC, in-depth experiments and clinical validation are still necessary.

## Figures and Tables

**Table 1:** Ingredient identification of QFD in vitro by UPLC-QTOF-MS.

No.	t_R_ (Min)	Identification (In Vitro)	Formula	[M+H]^+^ *m*/*z*			[M+Na]^+^ *m*/*z*	MS/MS Fragments	Source
				**Detected**	**Expected**	**Error (ppm)**			
1	2.691	karakolidine	C_22_H_35_NO_5_	394.2594	394.2593	0.3	416.2415	376.2487, 378.2635, 360.2529	FZ
2	3.321	chuanfumine	C_22_H_35_NO_5_	394.2597	394.2593	1.0	416.2391	376.2482, 358.2371	FZ
3	3.706	senbusine A	C_23_H_37_NO_6_	424.2706	424.2699	1.6	446.2698	406.2588, 388.2485	FZ
4	4.019	mesaconine	C_24_H_39_NO_9_	486.2704	486.2703	0.2	508.2523	436.2331, 404.2068, 454.2440	FZ
5	4.564	kaempferol	C_15_H_10_O_6_	287.0551	287.0550	0.3	309.0370	287.0549, 289.0609, 286.0467	HQ
6	4.799	16-β-hydroxycardiopetaline	C_21_H_33_NO_4_	364.2489	364.2488	0.3	386.2276	346.2380, 358.2378	FZ
7	4.912	(R)-norcoclaurine	C_16_H_17_NO_3_	272.1280	272.1281	−0.4	294.1101	136.0637, 282.1080	FZ
8	5.160	senbusine B	C_23_H_37_NO_6_	424.2700	424.2699	0.2	446.2505	406.2587, 388.2485	FZ
9	5.307	karakoline	C_22_H_35_NO_4_	378.2643	378.2639	1.1	400.2458	360.2541, 356.2220	FZ
10	5.476	isotalatizidine	C_23_H_37_NO_5_	408.2753	408.2750	0.7	430.2560	390.2640, 372.2533	FZ
11	5.634	aconine	C_25_H_41_NO_9_	500.2858	500.2860	−0.4	522.2730	450.2487, 468.2590	FZ
12	5.960	songorine	C_22_H_31_NO_3_	358.2382	358.2382	0	380.2220	340.2317, 342.2411	FZ
13	6.164	hetisine	C_20_H_27_NO_3_	330.2070	330.2069	0.3	352.1800	310.1799, 328.1909	FZ
14	6.638	hypaconine	C_24_H_39_NO_8_	470.2754	470.2754	0	492.2528	438.2487, 439.2519	FZ
15	7.044	fuziline	C_24_H_39_NO_7_	454.2808	454.2805	0.7	476.2640	404.2431, 436.2695	FZ
16	7.495	neoline	C_24_H_39_NO_6_	438.2852	438.2856	−0.9	460.2676	420.2749, 388.2482, 154.1226	FZ
17	7.540	14-acetylkarakoline	C_24_H_37_NO_5_	420.2754	420.2750	0.9	442.2834	388.2485, 102.0912, 402.2641	FZ
18	8.454	guan fu base H	C_22_H_33_NO_2_	344.2597	344.2590	2.0	366.1071	390.2643, 372.2532	FZ
19	8.476	talatisamine	C_24_H_39_NO_5_	422.2912	422.2906	1.4	444.2697	342.2427	FZ
20	9.435	14-acetyneoline	C_26_H_41_NO_7_	480.2964	480.2961	0.6	502.2722	462.2854, 331.0813	FZ
21	9.694	isorhamnetin	C_16_H_12_O_7_	317.0661	317.0656	1.6	339.0475	153.0191, 217.0489, 203.034	HQ
22	9.920	calycosin-7-O-β-D-glucoside	C_22_H_22_O_10_	447.1294	447.1291	0.7	469.1120	285.0758	HQ
23	10.551	14-acetyltalatizamine	C_26_H_41_NO_6_	464.3015	464.3012	0.6	486.2557	432.2746	FZ
24	11.323	karanjin	C_18_H_12_O_4_	293.0813	293.0808	1.7	315.0628	293.0797, 278.0583, 277.0468	FZ
25	13.590	benzoylmesaconitine	C_31_H_43_NO_10_	590.2967	590.2965	0.3	612.2778	540.2596, 558.2701	FZ
26	13.925	formononetin-O-β-D-glucoside	C_22_H_22_O_9_	431.1343	431.1342	0.2	453.1165	269.0806	HQ
27	14.209	calycosin-7-O-β-D-glc-6′′-O-acetate	C_24_H_24_O_11_	489.1403	489.1397	1.2	511.1214	371.2285	HQ
28	14.747	benzoylaconine	C_32_H_45_NO_10_	604.3128	604.3122	1.0	626.2895	554.2752, 572.2857	FZ
29	15.134	9,10-dimethoxy-pterocarpan-3-O-β-D-glucoside	C_23_H_26_O_10_	463.1606	463.1604	0.4	485.1432	299.0911, 160.0713	HQ
30	15.330	benzoylhypaconine	C_31_H_43_NO_9_	574.3022	574.3016	1.0	596.2847	542.2753, 570.3064	FZ
31	15.864	2′-hydroxy-3′,4′-dimethoxyisoflavan-7-O-β-D-glucoside	C_23_H_28_O_10_	465.1764	465.1761	0.6	487.1581	167.0700	HQ
32	16.090	calycosin	C_16_H_12_O_5_	285.0766	285.0763	1.0	307.0588	225.0546, 253.0495, 137.0230	HQ
33	17.024	benzoyldeoxyaconine	C_32_H_45_NO_9_	588.3177	588.3173	0.7	610.2787	556.2908	FZ
34	17.297	quercetin	C_15_H_10_O_7_	303.0506	303.0499	2.3	325.0319	257.0440, 285.0390, 247.0596	HQ
35	17.567	beiwudine	C_31_H_41_NO_8_	556.2909	556.2905	0.7	578.2724	524.2648	FZ
36	17.759	formononetin-7-O-β-D-glc-6′-β-O-acetate	C_24_H_24_O_10_	473.1449	473.1448	0.2	495.1262	270.0842, 139.1112	HQ
37	18.289	9,10-dimethoxy-pterocHQpan-3-O-β-D-glc-6′-O-acetate	C_25_H_28_O_11_	505.1714	505.1710	0.8	527.1530	487.3125, 311.2218	HQ
38	18.616	2′-hydroxy-3′,4′-dimethoxyisoflavan-7-O-β-D-glc-6″-O-acetate	C_25_H_30_O_11_	507.1871	507.1866	0.9	529.1675	442.2586, 167.0711	HQ
39	18.650	hypaconitine	C_33_H_45_NO_10_	616.3123	616.3122	0.2	638.7884	556.2898, 129.1019	FZ
40	19.406	formononetin	C_16_H_12_O_4_	269.0815	269.0814	0.4	291.0631	213.0909, 237.0544, 118.0411	HQ
41	19.541	isoastragalosideIV	C_41_H_68_O_14_	-	785.4687	-	807.4508	175.0597, 157.0491	HQ
42	19.620	astragaloside IV	C_41_H_68_O_14_	-	785.4687	-	807.4516	437.3402, 455.3499, 419.3302	HQ
43	20.116	7,2′-Dihydroxy-3′,4′-dimethoxyisoflavan	C_17_H_18_O_5_	303.1233	303.1232	0.3	325.1065	133.0644, 161.0594	HQ
44	20.442	soyasaponin I	C_48_H_78_O_18_	943.5264	943.5266	−0.2	965.5077	441.3721, 599.3967, 797.4655	HQ
45	20.898	astragaloside II	C_43_H_70_O_15_	827.4791	827.4793	−0.2	849.4624	669.3980, 453.3356	HQ
46	21.820	deoxyandrographolide	C_20_H_30_O_4_	335.2203	335.2217	−4.1	357.2036	263.1314, 247.1543	FZ
47	21.978	agroastragaloside III	C_51_H_82_O_21_	1031.5400	1031.5427	−2.0	1053.5288	898.4067, 900.4128	HQ
48	22.451	astragaloside I	C_45_H_72_O_16_	869.4886	869.4899	−1.5	891.4728	217.0704, 143.1065, 139.0386	HQ
49	22.674	isoastragaloside I	C_45_H_72_O_16_	869.4888	869.4899	−1.3	891.4739	217.0704, 143.1065, 157.0491	HQ
50	24.267	acetylastragaloside I	C_47_H_74_O_17_	911.5003	911.5004	−0.1	933.4817	143.1064, 199.0597	HQ

Abbreviations: HQ, Huangqi; FZ, Fuzi. The ‘-’ indicates that the M+H peak of the compound was not detected.

## Data Availability

The data presented in this study are available on request from the corresponding author. The data are not publicly available due to intellectual property protection.

## Supplementary Materials

The following supporting information can be downloaded at: https://www.mdpi.com/article/10.3390/ph18081109/s1, Figure S1: The base peak chromatogram (BPC) of QFD from UPLC-QTOF-MS in positive ion mode (A). The extracted ion chromatograms (EICs) of two representative components (songorine and astragaloside IV) in the mixed standard solution (B) and in the extracts of QFD (C); Figure S2: The OPLS-DA scores plots (A) and 200 permutation tests (B) from UPLC-QTOFMS dataset of myocardial samples in control and LPS groups. The OPLS-DA scores plots (C) and 200 permutation tests (D) from UPLC-QTOFMS dataset of serum samples in control and LPS groups; Figure S3: The PPI network of the intersecting genes between QFD and SIC constructed by string database; Table S1: Ingredient identification of QFD in vivo by UPLC-QTOF-MS; Table S2: Identification of differentiating metabolic features in cardiac tissue detected using UPLC-QTOF-MS; Table S3: Identification of differentiating metabolic features in serum detected using UPLC-QTOF-MS; Table S4: Active ingredients screened in Huangqi based on TCMSP database; Table S5: Active ingredients screened in Fuzi based on TCMSP database; Table S6: 26 core genes screened with centiscape plug-in in cytoscape; Table S7: Molecular docking results; Table S8 MM/GBSA binding energy.

## Abbreviations

CCK-8, cell counting kit-8; CK-MB, creatine kinase–myocardial band; COX2, cyclooxygenase 2; CPT, carnitine palmitoyltransferase; Dex, dexamethasone; FAO, fatty acid oxidation; GAPDH, glyceraldehyde 3-phosphate dehydrogenase; HE, hematoxylin–eosin; IL-1β/6, interleukin-1β/6; LDH, lactate dehydrogenase; LPS, lipopolysaccharide; LVEF, left ventricular ejection fraction; LVFS, left ventricular fractional shortening; LysoPC, lysophosphatidylcholine; NF-κB, nuclear factor kappa-B; NLRP3, nod-like receptor protein 3; NO, nitric oxide; OPLS-DA, orthogonal partial least squares discrimination analysis; PCA, principal component analysis; PLA2, phospholipase A2; PLS-DA, partial least squares discriminant analysis; PPARα, peroxisome proliferator activated receptor α; QFD, Qifu decoction; ROS, reactive oxygen species; SIC, sepsis-induced cardiomyopathy; TLR4, toll-like receptor 4; TNF-α, tumor necrotic factor-α.

## References

[1] Singer M., Deutschman C.S., Seymour C.W., Shankar-Hari M., Annane D., Bauer M., Bellomo R., Bernard G.R., Chiche J.-D., Coopersmith C.M. (2016). The Third International Consensus Definitions for Sepsis and Septic Shock (Sepsis-3). J. Am. Med. Assoc..

[2] Ravikumar N., Sayed M.A., Poonsuph C.J., Sehgal R., Shirke M.M., Harky A. (2021). Septic Cardiomyopathy: From Basics to Management Choices. Curr. Probl. Cardiol..

[3] Hollenberg S.M., Singer M. (2021). Pathophysiology of sepsis-induced cardiomyopathy. Nat. Rev. Cardiol..

[4] Wang L., Zhao Y., Su Z., Zhao K., Li P., Xu T. (2023). Ginkgolide A targets forkhead box O1 to protect against lipopolysaccharide-induced septic cardiomyopathy. Phytother. Res..

[5] Zhang G., Wang X., Li C., Li Q., An Y.A., Luo X., Deng Y., Gillette T.G., Scherer P.E., Wang Z.V. (2021). Integrated Stress Response Couples Mitochondrial Protein Translation with Oxidative Stress Control. Circulation.

[6] Pearce L., Davidson S.M., Yellon D.M. (2021). Does remote ischaemic conditioning reduce inflammation? A focus on innate immunity and cytokine response. Basic. Res. Cardiol..

[7] Shvilkina T., Shapiro N. (2023). Sepsis-Induced myocardial dysfunction: Heterogeneity of functional effects and clinical significance. Front. Cardiovasc. Med..

[8] Xu X., Liu Q., He S., Zhao J., Wang N., Han X., Guo Y. (2018). Qiang-Xin 1 Formula Prevents Sepsis-Induced Apoptosis in Murine Cardiomyocytes by Suppressing Endoplasmic Reticulum- and Mitochondria-Associated Pathways. Front. Pharmacol..

[9] Wang Y., Fu X., Shang Z., Qiao Y., Liu Y., Zhou L., Liu D. (2024). In vivo and in vitro study on the regulatory mechanism of XiaoChaiHu decoction on PANoptosis in sepsis-induced cardiomyopathy. J. Ethnopharmacol..

[10] Liang X.L., Ji M.M., Chen L., Liao Y., Kong X.Q., Xu X.Q., Liao Z.G., Wilson D.W. (2021). Traditional Chinese herbal medicine Astragalus Radix and its effects on intestinal absorption of aconite alkaloids in rats. Chin. Herb. Med..

[11] Fu J., Wang Z., Huang L., Zheng S., Wang D., Chen S., Zhang H., Yang S. (2014). Review of the botanical characteristics, phytochemistry, and pharmacology of Astragalus membranaceus (Huangqi). Phytother. Res..

[12] Zhou G., Tang L., Zhou X., Wang T., Kou Z., Wang Z. (2015). A review on phytochemistry and pharmacological activities of the processed lateral root of Aconitum carmichaelii Debeaux. J. Ethnopharmacol..

[13] Tan G., Jing J., Zhu Z., Lou Z., Li W., Zhao L., Zhang G., Chai Y. (2012). Detection and identification of diterpenoid alkaloids, isoflavonoids and saponins in Qifu decoction and rat plasma by liquid chromatography-time-of-flight mass spectrometry. Biomed. Chromatogr..

[14] Han Z., Guo L., Yu X., Guo H., Deng X., Yu J., Deng X., Xu F., Zhang Z., Huang Y. (2022). Network-driven targeted analysis reveals that Astragali Radix alleviates doxorubicin-induced cardiotoxicity by maintaining fatty acid homeostasis. J. Ethnopharmacol..

[15] Liu W., Zou X., Zheng Y., Zhang Y., Cui G., Liu S., Sun C., Peng C. (2025). Aconiti Lateralis Radix Praeparata ameliorates heart failure via PI3K/AKT/Bnip3 pathway. Front. Pharmacol..

[16] Li M., Han B., Zhao H., Xu C., Xu D., Sieniawska E., Lin X., Kai G. (2022). Biological active ingredients of Astragali Radix and its mechanisms in treating cardiovascular and cerebrovascular diseases. Phytomedicine.

[17] Su Z., Gao M., Weng L., Xu T. (2024). Esculin targets TLR4 to protect against LPS-induced septic cardiomyopathy. Int. Immunopharmacol..

[18] Zou X.Z., Hao J.F., Hou M.X. (2023). Hmgcs2 regulates M2 polarization of macrophages to repair myocardial injury induced by sepsis. Aging.

[19] Xia G., Shi H., Su Y., Han B., Shen C., Gao S., Chen Z., Xu C. (2022). Photoactivated adenylyl cyclases attenuate sepsis-induced cardiomyopathy by suppressing macrophage-mediated inflammation. Front. Immunol..

[20] Bougarne N., Weyers B., Desmet S.J., Deckers J., Ray D.W., Staels B., De Bosscher K. (2018). Molecular Actions of PPARα in Lipid Metabolism and Inflammation. Endocr. Rev..

[21] Wang M., Wang K., Liao X., Hu H., Chen L., Meng L., Gao W., Li Q. (2021). Carnitine Palmitoyltransferase System: A New Target for Anti-Inflammatory and Anticancer Therapy?. Front. Pharmacol..

[22] Mhetre N.M., Bhatambrekar A.L., Priya D., Saravanan V., Kathiravan M., Shevate K.S., Rajagopal K., Asgaonkar K.D., Chitre T.S. (2024). Rational design of some 1,3,4 trisubstituted pyrazole-thiazole derivatives to serve as MtInhA inhibitors using QSAR, ADMET, molecular docking, MM-GBSA, and molecular dynamics simulations approach. Chem. Phys. Impact.

[23] Charpentier J., Luyt C.E., Fulla Y., Vinsonneau C., Cariou A., Grabar S., Dhainaut J.F., Mira J.P., Chiche J.D. (2004). Brain natriuretic peptide: A marker of myocardial dysfunction and prognosis during severe sepsis. Crit. Care Med..

[24] Hochstadt A., Meroz Y., Landesberg G. (2011). Myocardial dysfunction in severe sepsis and septic shock: More questions than answers?. J. Cardiothorac. Vasc. Anesth..

[25] Sanfilippo F., Corredor C., Fletcher N., Landesberg G., Benedetto U., Foex P., Cecconi M. (2015). Diastolic dysfunction and mortality in septic patients: A systematic review and meta-analysis. Intensive Care Med..

[26] Pei X.B., Liu B. (2023). Research Progress on the Mechanism and Management of Septic Cardiomyopathy: A Comprehensive Review. Emerg. Med. Int..

[27] Lukić I., Mihić D., Varžić S.C., Relatić K.S., Zibar L., Loinjak D., Ćurić Ž.B., Klobučar L., Maričić L. (2024). Septic Cardiomyopathy. Rev. Cardiovasc. Med..

[28] Zhou W., Liu H., Qiu L.Z., Yue L.X., Zhang G.J., Deng H.F., Ni Y.H., Gao Y. (2021). Cardiac efficacy and toxicity of aconitine: A new frontier for the ancient poison. Med. Res. Rev..

[29] Cai Y., Gao Y., Tan G., Wu S., Dong X., Lou Z., Zhu Z., Chai Y. (2013). Myocardial lipidomics profiling delineate the toxicity of traditional Chinese medicine Aconiti Lateralis radix praeparata. J. Ethnopharmacol..

[30] Xue Z., Zhuo L., Zhang B., Zhu L., Xiang X., Zhang C., Liu W., Tan G., Liao W. (2023). Untargeted metabolomics reveals the combination effects and mechanisms of Huangqi-fuzi herb-pair against doxorubicin-induced cardiotoxicity. J. Ethnopharmacol..

[31] Chen X.S., Wang S.H., Liu C.Y., Gao Y.L., Meng X.L., Wei W., Shou S.T., Liu Y.C., Chai Y.F. (2022). Losartan attenuates sepsis-induced cardiomyopathy by regulating macrophage polarization via TLR4-mediated NF-κB and MAPK signaling. Pharmacol. Res..

[32] Sun L.J., Qiao W., Xiao Y.J., Cui L., Wang X., Ren W.D. (2019). Naringin mitigates myocardial strain and the inflammatory response in sepsis-induced myocardial dysfunction through regulation of PI3K/AKT/NF-kappaB pathway. Int. Immunopharmacol..

[33] Zhang T., Ma C., Zhang Z., Zhang H., Hu H. (2021). NF-kappaB signaling in inflammation and cancer. MedComm.

[34] Boaru S.G., Borkham-Kamphorst E., Van de Leur E., Lehnen E., Liedtke C., Weiskirchen R. (2015). NLRP3 inflammasome expression is driven by NF-κB in cultured hepatocytes. Biochem. Biophys. Res. Commun..

[35] Fang Z., Wang G., Huang R., Liu C., Yushanjiang F., Mao T., Li J. (2024). Astilbin protects from sepsis-induced cardiac injury through the NRF2/HO-1 and TLR4/NF-κB pathway. Phytother. Res..

[36] Luo M., Yan D., Sun Q., Tao J., Xu L., Sun H., Zhao H. (2020). Ginsenoside Rg1 attenuates cardiomyocyte apoptosis and inflammation via the TLR4/NF-kB/NLRP3 pathway. J. Cell. Biochem..

[37] Jiang L., Li X., Hu J., Tang Z. (2023). Mild Hypothermia Alleviates CLP-induced Multiple Organ Dysfunction by Mitigating Pyroptosis Through the TLR4/NF-κB/NLRP3 Signaling Pathway. Arch. Med. Res..

[38] Machado Dutra J., Espitia P.J.P., Andrade Batista R. (2021). Formononetin: Biological effects and uses—A review. Food Chem..

[39] Wang X., Li W., Zhang Y., Sun Q., Cao J., Tan N., Yang S., Lu L., Zhang Q., Wei P. (2022). Calycosin as a Novel PI3K Activator Reduces Inflammation and Fibrosis in Heart Failure Through AKT-IKK/STAT3 Axis. Front. Pharmacol..

[40] Stankovic S., Mutavdzin Krneta S., Djuric D., Milosevic V., Milenkovic D. (2025). Plant Polyphenols as Heart’s Best Friends: From Health Properties, to Cellular Effects, to Molecular Mechanisms of Action. Int. J. Mol. Sci..

[41] Mesaros C., Blair I.A. (2012). Targeted chiral analysis of bioactive arachidonic Acid metabolites using liquid-chromatography-mass spectrometry. Metabolites.

[42] Peretó J., López-García P., Moreira D. (2004). Ancestral lipid biosynthesis and early membrane evolution. Trends Biochem. Sci..

[43] Li C., Wang J., Wang Q., Zhang Y., Zhang N., Lu L., Wu Y., Zhang Q., Wang W., Wang Y. (2016). Qishen granules inhibit myocardial inflammation injury through regulating arachidonic acid metabolism. Sci. Rep..

[44] Niu Q.Y., Li Z.Y., Du G.H., Qin X.M. (2016). (1)H NMR based metabolomic profiling revealed doxorubicin-induced systematic alterations in a rat model. J. Pharm. Biomed. Anal..

[45] Wu D., Jian C., Peng Q., Hou T., Wu K., Shang B., Zhao M., Wang Y., Zheng W., Ma Q. (2020). Prohibitin 2 deficiency impairs cardiac fatty acid oxidation and causes heart failure. Cell Death Dis..

[46] Montaigne D., Butruille L., Staels B. (2021). PPAR control of metabolism and cardiovascular functions. Nat. Rev. Cardiol..

[47] Standage S.W., Bennion B.G., Knowles T.O., Ledee D.R., Portman M.A., McGuire J.K., Liles W.C., Olson A.K. (2017). PPARα augments heart function and cardiac fatty acid oxidation in early experimental polymicrobial sepsis. Am. J. Physiol. Heart Circ. Physiol..

[48] Zhu X.X., Wang X., Jiao S.Y., Liu Y., Shi L., Xu Q., Wang J.J., Chen Y.E., Zhang Q., Song Y.T. (2023). Cardiomyocyte peroxisome proliferator-activated receptor alpha prevents septic cardiomyopathy via improving mitochondrial function. Acta Pharmacol. Sin..

[49] Wang X.Y., Zhou Q.M., Guo L., Dai O., Meng C.W., Miao L.L., Liu J., Lin Q., Peng C., Xiong L. (2021). Cardioprotective effects and concentration-response relationship of aminoalcohol-diterpenoid alkaloids from *Aconitum carmichaelii*. Fitoterapia.

[50] Barrero M.J., Camarero N., Marrero P.F., Haro D. (2003). Control of human carnitine palmitoyltransferase II gene transcription by peroxisome proliferator-activated receptor through a partially conserved peroxisome proliferator-responsive element. Biochem. J..

[51] Fraser F., Corstorphine C.G., Zammit V.A. (1997). Topology of carnitine palmitoyltransferase I in the mitochondrial outer membrane. Biochem. J..

[52] Zeng K., Li Q., Song G., Chen B., Luo M., Miao J., Liu B. (2023). CPT2-mediated fatty acid oxidation inhibits tumorigenesis and enhances sorafenib sensitivity via the ROS/PPARγ/NF-κB pathway in clear cell renal cell carcinoma. Cell Signal..

[53] McCann M.R., George De la Rosa M.V., Rosania G.R., Stringer K.A. (2021). L-Carnitine and Acylcarnitines: Mitochondrial Biomarkers for Precision Medicine. Metabolites.

[54] Esser V., Brown N.F., Cowan A.T., Foster D.W., McGarry J.D. (1996). Expression of a cDNA isolated from rat brown adipose tissue and heart identifies the product as the muscle isoform of carnitine palmitoyltransferase I (M-CPT I). M-CPT I is the predominant CPT I isoform expressed in both white (epididymal) and brown adipocytes. J. Biol. Chem..

[55] Fukumoto K., Pierro A., Spitz L., Eaton S. (2002). Differential effects of neonatal endotoxemia on heart and kidney carnitine palmitoyl transferase I. J. Pediatr. Surg..

[56] Eaton S., Fukumoto K., Stefanutti G., Spitz L., Zammit V.A., Pierro A. (2003). Myocardial carnitine palmitoyltransferase I as a target for oxidative modification in inflammation and sepsis. Biochem. Soc. Trans..

[57] Chen X., Liu Y., Gao Y., Shou S., Chai Y. (2021). The roles of macrophage polarization in the host immune response to sepsis. Int. Immunopharmacol..

[58] Liu Y.C., Yu M.M., Shou S.T., Chai Y.F. (2017). Sepsis-Induced Cardiomyopathy: Mechanisms and Treatments. Front. Immunol..

[59] Ruan W., Ji X., Qin Y., Zhang X., Wan X., Zhu C., Lv C., Hu C., Zhou J., Lu L. (2021). Harmine Alleviated Sepsis-Induced Cardiac Dysfunction by Modulating Macrophage Polarization via the STAT/MAPK/NF-κB Pathway. Front. Cell Dev. Biol..

[60] Wang D., Lin Z., Zhou Y., Su M., Zhang H., Yu L., Li M. (2024). Atractylenolide I ameliorates sepsis-induced cardiomyocyte injury by inhibiting macrophage polarization through the modulation of the PARP1/NLRP3 signaling pathway. Tissue Cell.

[61] Shi X., Li T., Liu Y., Yin L., Xiao L., Fu L., Zhu Y., Chen H., Wang K., Xiao X. (2022). HSF1 Protects Sepsis-Induced Acute Lung Injury by Inhibiting NLRP3 Inflammasome Activation. Front. Immunol..

[62] Long C., Zhou Q., Xu M., Ding X., Zhang X., Zhang Y., Tang Y., Tan G. (2025). Sini decoction alleviates inflammation injury after myocardial infarction through regulating arachidonic acid metabolism. Chin. Herb. Med..

[63] Wang S., Tan K.S., Beng H., Liu F., Huang J., Kuai Y., Zhang R., Tan W. (2021). Protective effect of isosteviol sodium against LPS-induced multiple organ injury by regulating of glycerophospholipid metabolism and reducing macrophage-driven inflammation. Pharmacol. Res..

[64] Zhu H., Zhang L., Jia H., Xu L., Cao Y., Zhai M., Li K., Xia L., Jiang L., Li X. (2022). Tetrahydrocurcumin improves lipopolysaccharide-induced myocardial dysfunction by inhibiting oxidative stress and inflammation via JNK/ERK signaling pathway regulation. Phytomedicine.

[65] Yin L., Yuan L., Luo Z., Tang Y., Lin X., Wang S., Liang P., Huang L., Jiang B. (2024). COX-2 optimizes cardiac mitochondrial biogenesis and exerts a cardioprotective effect during sepsis. Cytokine.

