# Supplementary material for: Qifu Decoction Alleviates Lipopolysaccharide-Induced Myocardial Dysfunction by Inhibiting TLR4/NF-κB/NLRP3 Inflammatory Pathway and Activating PPARα/CPT Pathway
Source: Pharmaceuticals (Basel). 2025 Jul 25;18(8):1109. doi: 10.3390/ph18081109 (PMC12389671; doi:10.3390/ph18081109)
Supplement: Supplementary file 1 [file pharmaceuticals-18-01109-s001.zip › pharmaceuticals-3741033-supplementary.pdf]

Supplementary Information

Qifu decoction alleviates lipopolysaccharide-induced myocardial dysfunction by inhibiting TLR4/NF-κB/NLRP3 inflammation pathway and activating PPARα/CPT pathway

Ling-xin Zhuo[a,b], Ming-xuan Ma[a,b], Jia-yi Zhang[a,b], Jia-yu Zhou[a,b], Yu-qi Zheng[a,b], Ai-yin Liang[a,b], Qing-qing Sun[a,b], Jia Liu[c], Wen-ting Liao[a,b,*]

[a] *Department of Pharmaceutical Analysis, China Pharmaceutical University, Nanjing 210009, China*

[b] *Key Laboratory of Drug Quality Control and Pharmacovigilance, China Pharmaceutical University, Ministry of Education, Nanjing 210009, China*

[c] *Pharmic Laboratory Animal Center, China Pharmaceutical University, Nanjing 210009, China*

*Corresponding authors: Wenting Liao, Email: lwting84@163.com, Tel: +86-25-83271038.

Supplementary Methods

Ingredient identification of QFD in vitro

QFD were separated through Agilent 1290 Infinity II liquid chromatography system equipped with an ACQUITY UPLC HSS T3 column (2.1 × 100 mm, 1.8 μm) and detected with Agilent 6545 Quadrupole Time-of-Flight (Q-TOF) mass spectrometer. The mobile phase consisted of ultra-pure water-acetonitrile (95:5, v/v) containing 0.1% formic acid (A) and acetonitrile containing 0.1% formic acid (B). The elution gradient and instrument parameters were as follows: 0-8 min, 0-15% B; 8-12 min, 15%-20% B; 12-14 min, 20%-25% B; 14-16 min, 25%-30% B; 16-23 min, 30%-65% B; 23-28 min, 65-95% B; 28-30min, 95%-0% B; flow rate, 0.3 mL/min; injection volume, 5 μL; the column oven, 40 °C. The Q-TOF-MS was operated in full-scan MS resolution mode in positive ionization mode (ESI+). The instrument parameters were programmed as follows: mass range, 100-1200 m/z; fragmentor, 120 V; drying gas, 11 L/min; gas temp, 350ºC; spray pressure, 45psig; capillary voltage, 3.5 kV; collision energy, 25V.

The obtained typical total ion current (TIC) chromatogram were processed for peak extraction and peak matching using MassHunter Qualitative Analysis B.10.0 software, and the structures of the constituents were finally determined by comparing MS/MS spectra with the quasi-molecular ion peaks and fragmentation cleavage patterns of the compounds provided in the HMDB database on the basis of satisfying mass error ≤5 ppm and isotopic abundance ratio ≤5.

Ingredient identification of QFD in vivo

After 7 days of adaptive feeding, 7 SD rats were orally administered QFD at a dose of 30 g/kg. Blood samples were collected from the eye sockets at different time points before administration (0 min) and after administration (15, 30, 60, 120 min), with a volume of approximately 0.5 mL each time. The samples were collected in micro blood collection tubes containing 50 μL of heparin sodium, left to stand for 10 min, and then centrifuged at room temperature for 5 min at 5000 rpm to separate the plasma. 100 μL

of rat plasma was added to 3 times the volume of methanol, vortexed for 2 min, and centrifuged at 4 °C and 13000 rpm for 10 min to obtain the supernatant. The supernatant was dried under nitrogen flow, dissolved in 100 μL of 70% methanol, vortexed until dissolved, centrifuged at 4 °C and 13000 rpm for 10 min, and 80-100 μL of the supernatant was taken and added to an injection vial. Chromatography and mass spectrometry conditions were the same as in vitro identification.

Supplementary Figures

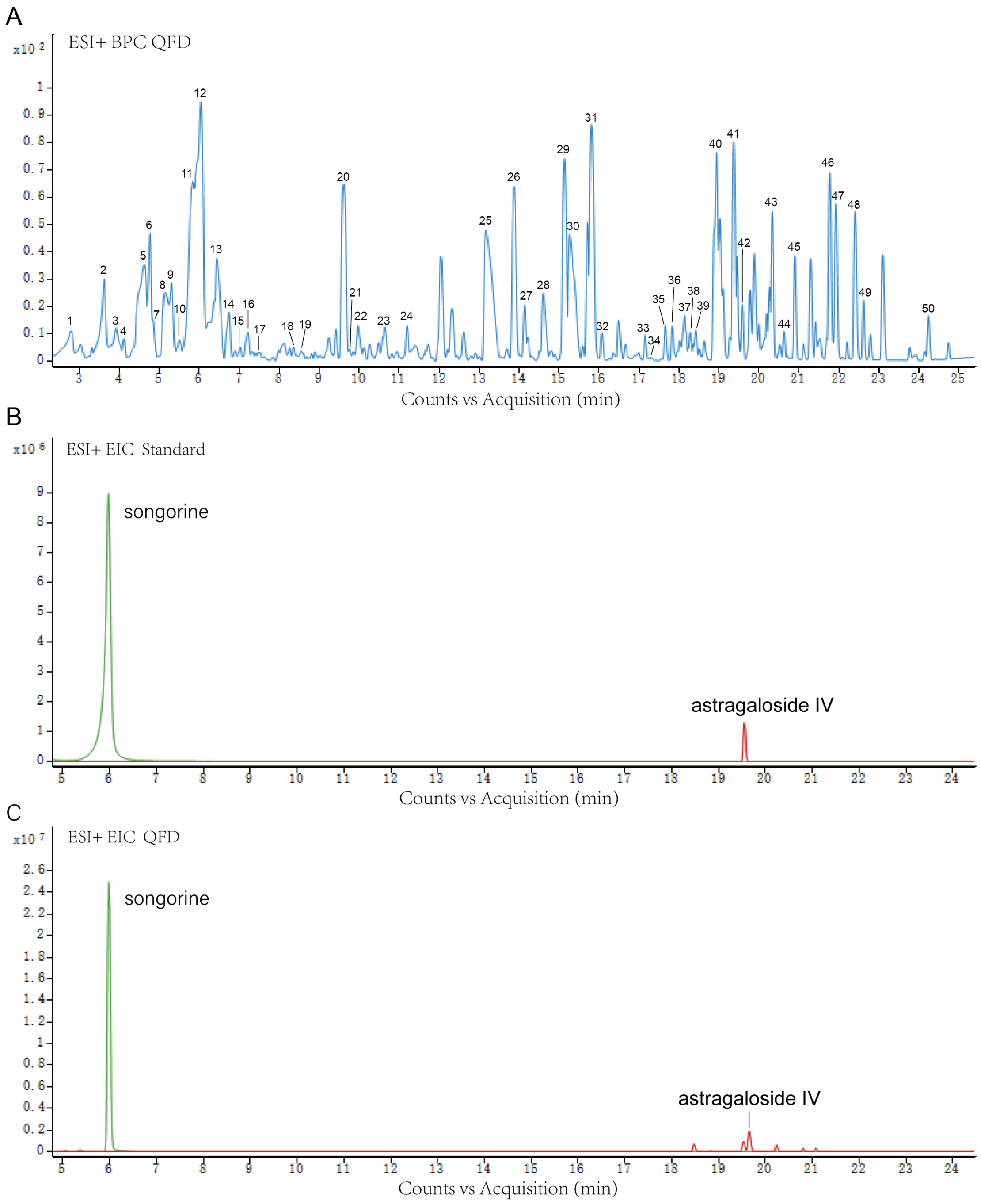

Figure S1. The base peak chromatogram (BPC) of QFD from UPLC-QTOF-MS in positive ion mode (A). The extracted ion chromatograms (EICs) of two representative components (songorine and astragaloside IV) in the mixed standard solution (B) and in the extracts of QFD (C).

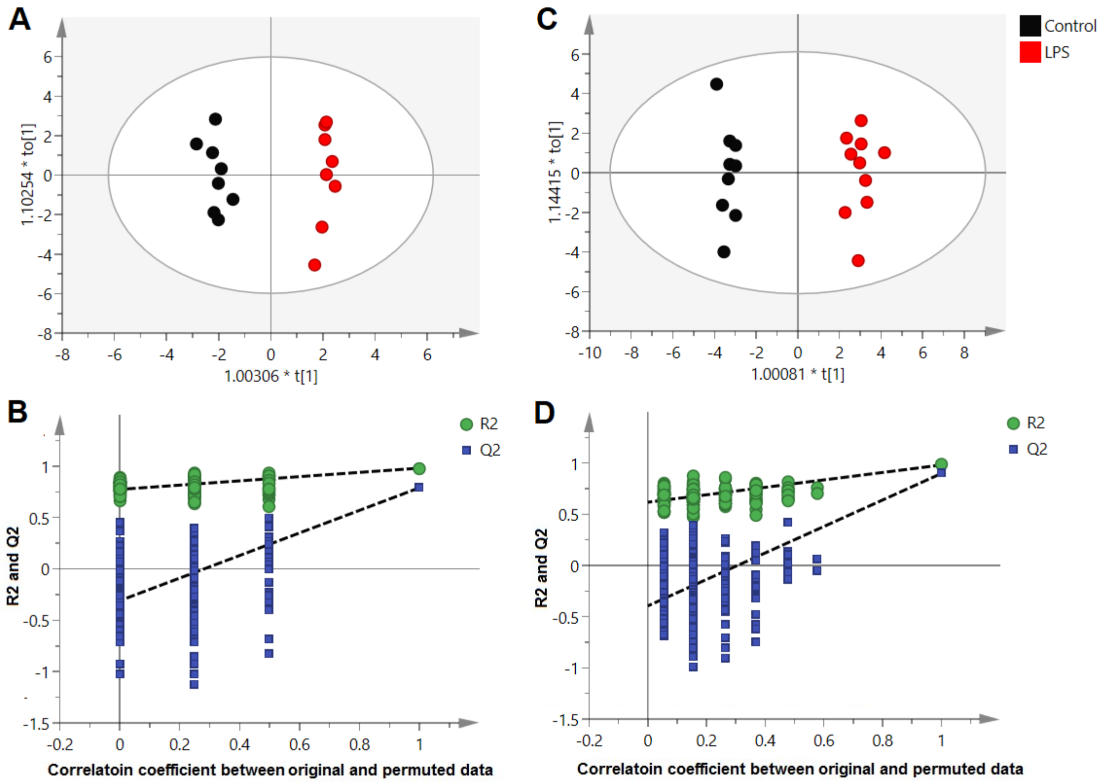

Figure S2. The OPLS-DA scores plots (A) and 200 permutation tests (B) from UPLC-QTOFMS dataset of myocardial samples in control and LPS groups. The OPLS-DA scores plots (C) and 200 permutation tests (D) from UPLC-QTOFMS dataset of serum samples in control and LPS groups.

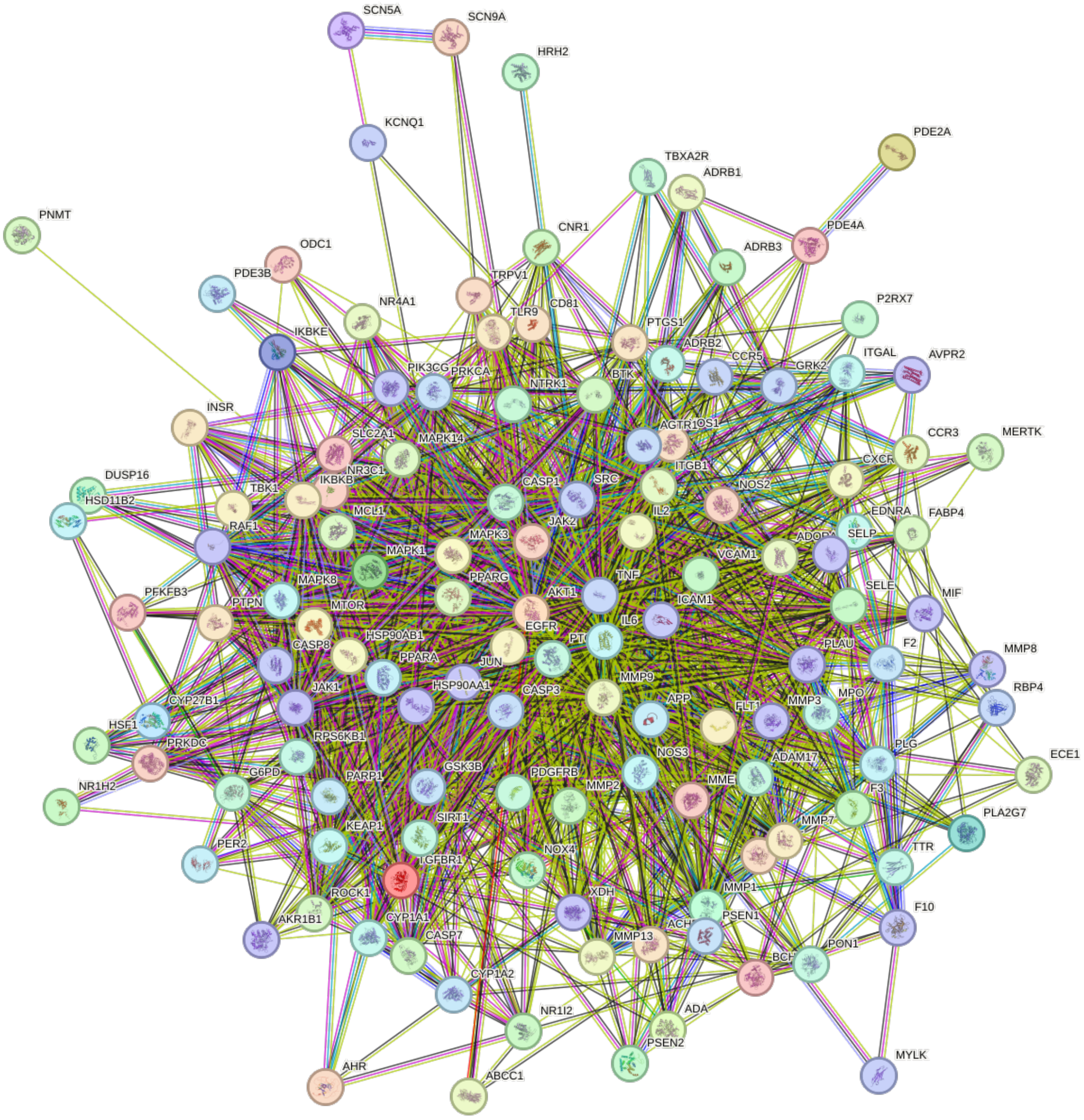

Figure S3. The PPI network of the intersecting genes between QFD and SIC constructed by string database.

Supplementary Tables

Table S1 Ingredient identification of QFD in vivo by UPLC-QTOF-MS.

No.	t_R (min)	Identification (in vivo)	Formula	$[M+H]^+$ m/z			$[M+Na]^+$ m/z	MS/MS fragments
				detected	expected	error (ppm)		
1	1.46	Dehydration isotalatizidine*	$C_{23}H_{35}NO_4$	390.2247	390.2244	0.8	412.2458	372.1002
2	2.57	Karakolidine	$C_{22}H_{35}NO_5$	394.2590	394.2593	-0.8	416.2415	376.2487, 378.2635, 360.2529
3	3.48	Demethyl neoline*	$C_{23}H_{37}NO_6$	424.2695	424.2699	-0.9	416.2391	406.2196, 374.2332
4	3.67	Chuanfumine	$C_{22}H_{35}NO_5$	394.2589	394.2593	-1.0	416.2391	376.2482, 358.2371
5	4.15	Mesaconine	$C_{24}H_{39}NO_9$	486.2702	486.2703	-0.2	508.2523	436.2331, 404.2068, 454.2440
6	4.43	16-β-hydroxycardiopetaline	$C_{21}H_{33}NO_4$	364.2487	364.2488	-0.3	386.2276	346.2380, 358.2378
7	4.71	Senbusine B	$C_{23}H_{37}NO_6$	424.2691	424.2699	-1.9	446.2505	406.2587, 388.2485
8	4.73	Demethyl dehydrogen isatalatizidine*	$C_{22}H_{33}NO_5$	392.2435	392.2437	-0.5	414.2251	374.0973, 360.2126
9	4.92	Demethyl karakolidine*	$C_{21}H_{33}NO_5$	380.2436	380.2437	-0.3	402.2251	362.2304, 344.0126
10	4.99	Karakoline	$C_{22}H_{35}NO_4$	378.2641	378.2639	0.5	400.2458	360.2541, 356.2220
11	5.05	Isotalatizidine	$C_{23}H_{37}NO_5$	408.2747	408.2750	-0.7	430.2560	390.2640, 372.2533
12	5.39	Aconine	$C_{25}H_{41}NO_9$	500.2856	500.2860	-0.8	522.2730	450.2487, 468.2590
13	5.51	Songorine	$C_{22}H_{31}NO_3$	358.2388	358.2382	1.7	380.2220	340.2317, 342.2411
14	5.55	Dehydration karakoline*	$C_{22}H_{33}NO_3$	360.2536	360.2540	-1.1	382.2353	348.2142, 330.1795
15	5.73	Hetisine	$C_{20}H_{27}NO_3$	330.2065	330.2069	-1.2	352.1800	310.1799, 328.1909
16	6.13	Demethyl dehydrogen neoline*	$C_{23}H_{35}NO_6$	422.2542	422.2543	-0.2	444.2357	404.2442, 372.1027
17	6.19	Demethyl neoline*	$C_{23}H_{37}NO_6$	424.2700	424.2699	0.2	446.2513	374.0923
18	6.26	Hypaconine	$C_{24}H_{39}NO_8$	470.2750	470.2754	-0.9	492.2528	438.2487, 439.2519
19	6.37	Fuziline	$C_{24}H_{39}NO_7$	454.2804	454.2805	-0.2	476.2640	404.2431, 436.2695
20	6.90	Neoline	$C_{24}H_{39}NO_6$	438.2854	438.2856	-0.5	460.2676	420.2749, 388.2482, 154.1226
21	7.97	Talatisamine	$C_{24}H_{39}NO_5$	422.2908	422.2906	0.5	444.2697	390.2643, 372.2532
22	8.95	14-acetyneoline	$C_{26}H_{41}NO_7$	480.2956	480.2961	-1.0	502.2722	462.2854, 331.0813
23	9.39	Calycosin-7-O-β-D-glucoside	$C_{22}H_{22}O_{10}$	447.1292	447.1291	0.2	469.1120	285.0758
24	10.18	14-acetyltalatizamine	$C_{26}H_{41}NO_6$	464.3010	464.3012	-0.4	486.2557	432.2746

25	11.63	Karanjin	$C_{18}H_{12}O_4$	293.0812	293.0808	1.4	315.0628	293.0797, 278.0583, 277.0468
26	13.77	Formononetin-O-glucuronide*	$C_{22}H_{20}O_{10}$	445.1131	445.1135	-0.9	467.0949	267.2670, 252.1558
27	15.44	Calycosin-O-glucuromide*	$C_{22}H_{20}O_{11}$	461.1085	461.1084	0.2	483.0898	285.0756, 270.0504
28	15.75	Calycosin	$C_{16}H_{12}O_5$	285.0760	285.0763	-1.1	307.0588	225.0546, 253.0495, 137.0230
29	18.70	Hypaconitine	$C_{33}H_{45}NO_{10}$	616.3117	616.3122	-0.8	638.7884	556.2898, 129.1019
30	19.23	Formononetin	$C_{16}H_{12}O_4$	269.0810	269.0814	-1.5	291.0631	213.0909, 237.0544, 118.0411

The compounds marked with “*” indicate metabolic products.

Table S2 Identification of differentiating metabolic features in cardiac tissue detected using UPLC-QTOF-MS.

RT (min)	Adduct	Detected mass (m/z)	Theoretical mass (m/z)	Delta (ppm)	MS/MS fragments	Metabolites[a]	VIP[c]	Control/LPS		QFD-H/LPS	
								Ratio	*p* value	Ratio	*p* value
0.696	M+H	613.1587	613.1592	1	484.13, 409.29, 355.19	*Oxidized glutathione*[b]	1.04	1.35	1.99E-02	1.42	6.58E-03
0.718	M+H	134.0448	134.0448	0	118.92, 110.03, 88.04	*L-Aspartic acid*[b]	1.36	4.03	9.62E-03	2.53	3.23E-02
0.721	M+H	248.1485	248.1492	3	85.03, 60.08	3-Hydroxybutyrylcarnitine	4.21	2.41	9.69E-04	1.57	5.03E-01
0.733	M+H	308.0904	308.0911	2	290.27, 233.09, 179.07	*Glutathione*[b]	2.83	1.73	3.57E-02	1.64	4.75E-02
0.759	M+H	284.0987	284.0989	1	152.04, 135.02, 110.03	Guanosine	1.03	1.53	1.15E-02	0.95	1.00E+00
0.780	M+H	268.1040	268.1040	0	136.06	*Adenosine*[b]	13.75	2.51	1.32E-04	1.71	1.88E-02
0.780	M+H	136.0618	136.0618	0	119.04, 110.03, 81.94	*Adenine*[b]	6.36	2.08	3.05E-03	1.59	3.84E-02
0.882	M+H	132.1020	132.1019	1	86.06, 56.97	L-Isoleucine	7.33	1.29	2.15E-02	1.10	9.66E-01
1.150	M+H	209.0913	209.0921	4	192.06, 120.04, 94.06	L-Kynurenine	1.02	0.53	1.85E-04	0.98	1.00E+00
5.782	M+H	316.2487	316.2482	2	257.18, 155.14, 85.03, 60.08	*Decanoylcarnitine*[b]	2.89	3.32	7.29E-05	2.79	1.04E-02
6.447	M+H	368.2799	368.2795	1	85.03, 60.08	*3, 5-Tetradecadiencarnitine*[b]	3.02	4.15	3.83E-05	3.44	3.15E-03
6.545	M+H	344.2801	344.2795	2	285.21, 183.17, 85.03, 60.08	*Dodecanoylcarnitine*[b]	4.43	5.38	5.66E-07	3.57	4.63E-03
6.995	M+H	370.2956	370.2952	1	311.22, 144.10, 85.03, 60.08	*Tetradecenoylcarnitine*[b]	4.81	5.54	3.87E-06	4.87	5.32E-05
7.318	M+H	396.3113	396.3108	1	85.03, 60.08	*9,12-Hexadecadienoylcarnitine*[b]	7.21	6.82	4.22E-05	4.55	9.30E-03
7.710	M+H	372.3112	372.3108	1	313.24, 142.16, 85.03, 60.08	*Tetradecanoylcarnitine*[b]	9.34	5.94	1.06E-08	3.15	1.38E-03
7.927	M+H	302.3057	302.3054	1	284.29, 106.09, 88.08, 57.07	Sphinganine	2.88	0.60	5.08E-06	0.96	1.00E+00
8.126	M+H	398.3268	398.3265	1	339.25, 144.10, 85.03, 60.08	*9-Hexadecenoylcarnitine*[b]	8.36	6.42	5.86E-07	3.72	3.76E-03
8.509	M+H	424.3424	424.3421	1	263.23, 144.10, 85.03, 60.08	*Linoleyl carnitine*[b]	10.82	7.14	2.97E-08	4.67	8.30E-05
9.174	M+H	400.3429	400.3421	2	144.10, 85.03, 60.08	*Palmitoylcarnitine*[b]	14.52	5.73	5.25E-06	3.06	3.31E-02
9.498	M+H	426.3579	426.3578	0	367.28, 144.10, 85.03, 60.08	*Oleoylcarnitine*[b]	18.56	6.99	3.37E-06	4.45	3.16E-03
9.939	M+H	414.3556	414.3578	5	85.03, 60.08	*Heptadecanoyl carnitine*[b]	2.40	6.02	1.90E-06	3.16	8.98E-03
10.687	M+H	428.3733	428.3734	0	369.30, 85.03	*Stearoylcarnitine*[b]	11.31	3.90	5.64E-06	2.07	1.23E-02
12.337	M+H	852.5517	852.5538	2	184.07	*PC(22:6)/20:5)*[b]	5.86	2.26	5.36E-03	1.77	2.85E-02

[a] The metabolites marked with "[b]" in italics were significantly reversed by QFD.

[c] Variable importance in the projection (VIP) was obtained from the OPLS-DA model.

Table S3 Identification of differentiating metabolic features in serum detected using UPLC-QTOF-MS.

RT (min)	Adduct	Detected mass (m/z)	Theoretical mass (m/z)	Delta (ppm)	MS/MS fragments	Metabolites[a]	VIP[c]	Control/LPS		QFD-H/LPS	
								Ratio	P value	Ratio	P value
1.15	M+H	160.0761	160.0757	2	143.04, 113,96, 56.94	Indoleacetaldehyde	3.16	2.00	7.42E-05	0.90	1.00E+00
1.48	M+H	209.0913	209.0921	4	192.06, 120.04, 94.06	L-Kynurenine	1.08	0.49	7.23E-03	0.95	6.89E-01
7.21	M+H	302.3059	302.3054	2	284.29, 106.09, 88.08, 57.07	*Sphinganine*[b]	6.02	1.67	4.62E-03	1.74	5.78E-04
7.26	M+H	380.2565	380.2560	1	264.27, 247.24	Sphingosine 1-phosphate	1.58	2.37	6.09E-11	1.17	3.81E-01
7.72	M+H	300.2901	300.2897	1	282.27, 252.27, 57.07	*Sphingosine*[b]	1.11	1.82	2.34E-04	1.53	2.26E-03
7.98	M+H	520.3399	520.3398	0	184.07, 104.11	*LysoPC(18:2)*[b]	4.74	1.68	7.89E-04	1.37	2.97E-02
8.18	M+H	568.3400	568.3398	0	184.07, 104.11	*LysoPC(22:6)*[b]	5.40	1.51	9.23E-03	1.50	1.96E-03
8.25	M+H	544.3400	544.3398	0	184.07, 104.11	LysoPC(20:4)	7.10	2.00	1.44E-04	1.43	1.51E-01
8.56	M+H	496.3397	496.3398	0	184.07, 104.11	*LysoPC(16:0)*[b]	7.24	1.82	1.69E-06	1.30	4.96E-02
8.69	M+H	424.3427	424.3421	1	263.23, 144.10, 85.03, 60.08	Linoleyl carnitine	1.90	1.66	1.02E-03	0.99	1.00E+00
9.31	M+H	522.3555	522.3554	0	184.07, 104.11	LysoPC(18:1)[b]	5.77	1.63	2.90E-04	1.33	4.32E-02
9.68	M+H	426.3581	426.3578	1	367.28, 144.10, 85.03, 60.08	Oleoylcarnitine	2.35	1.59	3.38E-02	1.00	1.00E+00
10.71	M+H	524.3721	524.3711	2	184.07, 104.11	*LysoPC(18:0)*[b]	10.17	1.78	1.59E-05	1.30	4.02E-02
11.29	M+H	335.2948	335.2945	1	321.32, 99.08, 89.06	*Docosatrienoic acid*[b]	1.35	0.33	1.59E-08	0.54	7.43E-06
12.24	M+H	305.2477	305.2475	1	287.24, 235.17, 221.15	*Arachidonic acid*[b]	1.33	1.59	1.66E-02	1.53	2.95E-02
14.80	M+H	703.5746	703.5749	0	184.07	SM(d18:1/16:0)	5.80	0.55	6.78E-03	1.02	1.00E+00

[a] The metabolites marked with "[b]" in italics were significantly reversed by QFD.

[c] Variable importance in the projection (VIP) was obtained from the OPLS-DA model.

Table S4 Active ingredients screened in Huangqi based on TCMSP database.

No.	Mol ID	Molecule Name	Formula	MW	OB (%)	DL
1	MOL000211	mairin	$C_{30}H_{48}O_3$	456.78	55.38	0.78
2	MOL000239	jaranol	$C_{17}H_{14}O_6$	314.31	50.83	0.29
3	MOL000296	hederagenin	$C_{30}H_{48}O_4$	414.79	36.91	0.75
4	MOL000033	(3S,8S,9S,10R,13R,14S,17R)-10,13-dimethyl-17-[(2R,5S)-5-propan-2-yloctan-2-yl]-2,3,4,7,8,9,11,12,14,15,16,17-dodecahydro-1H-cyclopenta[a]phenanthren-3-ol	$C_{30}H_{52}O$	428.82	36.23	0.78
5	MOL000354	isorhamnetin	$C_{16}H_{12}O_7$	316.28	49.6	0.31
6	MOL000371	3,9-di-O-methylnissolin	$C_{18}H_{18}O_5$	314.36	53.74	0.48
7	MOL000378	7-O-methylisomucronulatol	$C_{18}H_{20}O_5$	316.38	74.69	0.30
8	MOL000379	9,10-dimethoxypterocarpan-3-O-β-D-glucoside	$C_{23}H_{26}O_{10}$	462.49	36.74	0.92
9	MOL000380	(6aR,11aR)-9,10-dimethoxy-6a,11a-dihydro-6H-benzofurano[3,2-c] chromen-3-ol	$C_{17}H_{16}O_5$	300.33	64.26	0.42
10	MOL000387	bifendate	$C_{20}H_{18}O_{10}$	418.38	31.1	0.67
11	MOL000392	formononetin	$C_{16}H_{12}O_4$	268.28	69.67	0.21
12	MOL000398	isoflavanone	$C_{15}H_{12}O_2$	316.33	109.99	0.30
13	MOL000417	calycosin	$C_{16}H_{12}O_5$	284.28	47.75	0.24
14	MOL000422	kaempferol	$C_{15}H_{10}O_6$	286.25	41.88	0.24
15	MOL000433	FA	$C_{19}H_{19}N_7O_6$	441.45	68.96	0.71
16	MOL000438	(3R)-3-(2-hydroxy-3,4-dimethoxyphenyl) chroman-7-ol	$C_{17}H_{18}O_5$	302.35	67.67	0.26
17	MOL000098	quercetin	$C_{15}H_{10}O_7$	302.25	46.43	0.28

* Screening criteria were oral bioavailability（OB）≥ 30% and drug-like properties（DL）≥ 0.18.

Table S5 Active ingredients screened in Fuzi based on TCMSP database.

No.	Mol ID	Molecule Name	Formula	MW	OB (%)	DL
1	MOL002211	11,14-eicosadienoic acid	$C_{20}H_{36}O_2$	308.56	39.99	0.20
2	MOL002388	Delphin_qt	$C_{15}H_{11}O_7$	303.26	57.76	0.28
3	MOL002392	Deltoin	$C_{19}H_{20}O_5$	328.39	46.69	0.37
4	MOL002395	Deoxyandrographolide	$C_{20}H_{30}O_4$	334.5	56.3	0.31
5	MOL002397	karakoline	$C_{22}H_{35}NO_4$	377.58	51.73	0.73
6	MOL002398	Karanjin	$C_{18}H_{12}O_4$	292.3	69.56	0.34
7	MOL002401	Neokadsuranic acid B	$C_{30}H_{44}O_3$	452.74	43.1	0.85
8	MOL002410	benzoylnapelline	$C_{29}H_{37}NO_4$	463.67	34.06	0.53
9	MOL002415	6-Demethyldesoline	$C_{24}H_{39}NO_7$	453.64	51.87	0.66
10	MOL002419	(R)-Norcoclaurine	$C_{16}H_{17}NO_3$	271.34	82.54	0.21
11	MOL002421	ignavine	$C_{27}H_{31}NO_5$	449.59	84.08	0.25
12	MOL002434	Carnosifloside I_qt	$C_{30}H_{48}O_3$	456.78	38.16	0.80
13	MOL000359	sitosterol	$C_{29}H_{50}O$	414.79	36.91	0.75

* Screening criteria were oral bioavailability（OB）≥ 30% and drug-like properties（DL）≥ 0.18.

Table S6 26 core genes screened with centiscape plug-in in cytoscape.

Gene name	Degree unDir	Betweenness unDir	Closeness unDir
IL6	103	1672.90419	0.006410256
TNF	100	1402.850808	0.006289308
AKT1	93	1528.344789	0.006024096
CASP3	78	542.5780721	0.005494505
SRC	74	796.2441462	0.005376344
MAPK3	73	507.4242526	0.005347594
JUN	73	413.369903	0.005347594
MMP9	72	300.0595481	0.005319149
EGFR	70	356.5795253	0.005263158
PPARg	70	878.5475965	0.005291005
PTGS2	69	316.5032306	0.005235602
ICAM1	64	265.9726231	0.005050505
HSP90AA1	59	320.3086295	0.004975124
APP	54	317.7373019	0.004854369
IL2	54	162.0170583	0.004830918
HSP90AB1	53	241.1030758	0.004830918
GSK3B	52	195.1184729	0.004807692
VCAM1	52	123.8130501	0.004784689
SIRT1	48	181.8429105	0.004672897
CASP1	48	138.0957319	0.004651163
MAPK1	46	138.4734073	0.00462963
NOS3	44	142.7097062	0.004608295
PPARα	44	198.3900359	0.00462963
JAK2	43	128.30479	0.004545455
NR3C1	36	400.9767988	0.004464286
AGTR1	35	238.8987179	0.004385965

* Screening criteria were closeness undir＞0.004, betweenness undir＞117.71, and degree undir＞27.2.

Table S7 Molecular docking results.

Compound	TLR4	PPARα
Formononetin	-7.6	-7.9
Calycosin	-7.5	-7.8
Quercetin	-7.0	-8.5
Kaempferol	-7.5	-8.6
Isorhamnetin	-7.0	-8.4
（R）-Norcoclaurine	-6.9	-9.2
Karanjin	-7.7	-7.4
Deoxyandrographolide	-5.4	-5.4
karakoline	-6.1	-2.3

Table S8 MM/GBSA binding energy

	MM/GBSA dG bind TLR4	MM/GBSA dG bind PPARα
Formononetin	-47.23	-48.38
Calycosin	-38.69	-44.37
Quercetin	-41.80	-46.86
Kaempferol	-33.34	-42.84
Isorhamnetin	-38.66	-49.05
(R)-Norcoclaurine	-51.06	-51.90
Karanjin	-48.52	-43.47
Deoxyandrographolide	-43.31	\
Karakoline	-36.96	\
